# *Penicillium echinulatum* secretome analysis reveals the fungi potential for degradation of lignocellulosic biomass

**DOI:** 10.1186/s13068-016-0476-3

**Published:** 2016-03-17

**Authors:** Willian Daniel Hahn Schneider, Thiago Augusto Gonçalves, Cristiane Akemi Uchima, Matthew Brian Couger, Rolf Prade, Fabio Marcio Squina, Aldo José Pinheiro Dillon, Marli Camassola

**Affiliations:** Enzymes and Biomass Laboratory, Institute of Biotechnology, University of Caxias do Sul, Francisco Getúlio Vargas Street 1130, Caxias Do Sul, RS 95070-560 Brazil; Laboratório Nacional de Ciência e Tecnologia do Bioetanol (CTBE), Centro Nacional de Pesquisa em Energia e Materiais (CNPEM), Giuseppe Maximo Scolfaro 10.000, Campinas, São Paulo 13083-970 Brazil; Department of Biochemistry, Institute of Biology, State University of Campinas (UNICAMP), Campinas, São Paulo Brazil; Department of Microbiology and Molecular Genetics, Oklahoma State University, 1110 South Innovation Way, Stillwater, OK 74078 USA

**Keywords:** Lignocellulosic biomass, Biofuels, *Penicillium echinulatum* strains, Secretome, CAZymes

## Abstract

**Background:**

The enzymatic degradation of lignocellulosic materials by fungal enzyme systems has been extensively studied due to its effectiveness in the liberation of fermentable sugars for bioethanol production. Recently, variants of the fungus *Penicillium echinulatum* have been described as a great producer of cellulases and considered a promising strain for the bioethanol industry.

**Results:**

*Penicillium echinulatum,* wild-type 2HH and its mutant strain S1M29, were grown on four different carbon sources: cellulose, sugar cane bagasse pretreated by steam explosion (SCB), glucose, and glycerol for 120 h. Samples collected at 24, 96, and 120 h were used for enzymatic measurement, and the 96-h one was also used for secretome analysis by 1D-PAGE LC–MS/MS. A total of 165 proteins were identified, and more than one-third of these proteins belong to CAZy families. Glycosyl hydrolases (GH) are the most abundant group, being represented in larger quantities by GH3, 5, 17, 43, and 72. Cellobiohydrolases, endoglucanases, β-glycosidases, xylanases, β-xylosidases, and mannanases were found, and in minor quantities, pectinases, ligninases, and amylases were also found. Swollenin and esterases were also identified.

**Conclusions:**

Our study revealed differences in the two strains of *P. echinulatum* in several aspects in which the mutation improved the production of enzymes related to lignocellulosic biomass deconstruction. Considering the spectral counting analysis, the mutant strain S1M29 was more efficient in the production of enzymes involved in cellulose and hemicellulose degradation, despite having a nearly identical CAZy enzymatic repertoire. Moreover, S1M29 secretes more quantities of protein on SCB than on cellulose, relevant information when considering the production of cellulases using raw materials at low cost. Glucose, and especially glycerol, were used mainly for the production of amylases and ligninases.

**Electronic supplementary material:**

The online version of this article (doi:10.1186/s13068-016-0476-3) contains supplementary material, which is available to authorized users.

## Background

One of the global priorities is the development of alternative energy sources other than fossil fuels, and the use of cellulosic biomass has the potential to contribute to meeting the demand for liquid fuel [1]. Lignocellulose is the most abundant natural material present on earth and is composed of the polyphenol lignin and three polysaccharides: cellulose, hemicellulose, and pectin [2, 3]. These components are strongly intermeshed and chemically bonded by noncovalent forces and covalent crosslinkages, forming an intricately linked network that provides strength to the plant cell wall [4].

The manufacturing processes of saccharification and bioproducts from lignocellulose biomass are complex and lengthy ones [5]. One approach to depolymerization of plant cell wall polysaccharides involves using hydrolytic enzymes produced by some species of bacteria and fungi [6]. The degradation of lignocellulosic materials into monomeric sugars is of major importance, since the fermentable sugars can be used as raw materials in many biotechnological processes, including the production of second-generation ethanol [7]. In nature, fungi perform a major role in the degradation of plant biomass, producing an extensive set of a carbohydrate degrading enzyme specifically dedicated to break down plant cell wall polysaccharides. However, these varied sets differ between the species of fungi and the carbon sources employed. For example, while *Trichoderma reesei* has a set of highly efficient enzymes in the degradation of cellulose [8, 9], *Aspergillus* species produce many enzymes for degrading pectin [10].

*Penicillium* species have been reported to produce enzyme systems with higher performance than *T. reesei* and *A. niger* [11–14]. Among the species of *Penicillium*, *P. echinulatum* has been the focus of attention because of its potential to produce high titers of cellulases and has been considered a promising strain for the industry of second-generation ethanol [15–22]. All the mutant strains used for the production of cellulases are derived from the wild type called 2HH that was isolated from larvae of coleoptera *Anobium punctatum*. A mutant strain of *P. echinulatum* 9A02S1 was obtained from the strain 2HH, after several steps of mutagenesis, characterized by being a mutant partially depressed by glucose. This microorganism is deposited in the Deutsche Sammlung von Mikroorganismen und Zellkulturen–DSM 18942. The mutant strain S1M29 of *P. echinulatum* was obtained from the 9A02S1 strain through employing hydrogen peroxide mutagenesis and a selection of mutants in a medium supplemented with 2-deoxyglucose [15]. This mutant S1M29, due to high production of cellulases, is an industrial strain with many patents pending for approval.

In this context, it is important to study the secretome of fungal strains, since the genetic improvement process of these organisms are unknown. In this paper, we strived to elucidate the mechanism involved in S1M29-enhanced biomass decomposition phenotype on this question through the secretome level, presenting the first study of wild-type 2HH and mutant S1M29 secretomes of *P. echinulatum* in submerged cultivation using sugar cane bagasse pretreated by steam explosion (SCB), using cellulose, glucose, and glycerol as carbon sources. To supplement this study, an enzymatic analysis using a set of substrates was conducted in order to further validate and describe the enzymatic repertoire available to degrade lignocellulosic biomass by *P. echinulatum.*

## Results

### General features of the *P. echinulatum* genome strains

The fungus *P. echinulatum* has a genome with a similar size to other *Penicillium* species described in the literature [23, 24]. The genomic sequencing showed that both strains of *P. echinulatum*, wild-type 2HH and mutant S1M29, have a genome of the same size (Table 1). There were no significant changes in genome size with respect to the deletions in regions encoding proteins. Thus, changes in enzyme production between the two strains is probably explained at the transcription level and is not due to large scale genomic rearrangements.

**Table 1:** General features of *Penicillium echinulatum* genome

Category	Wild-type 2HH	Mutant S1M29
Genome statistic		
Genome size (MB)	29.82	29.83
Number of bases	29.821.618	29.827.032
Number of ambiguous bases (N)	92.041	82.971
Number of contigs	1018	1124
n50	147,886	137,718
n90	32,016	32,016
GC %	50.5	50.5
Number of predicted genes	8504	8552
Average gene size	1686	1684
Median gene size	1441	1442
Average protein size (AA)	505	504
Number of predicted exons	25.098	25.494
Number of predicted introns	16,638	16,997
Average number of exons per gene	3	3
Average number of introns per gene	2	2
Average exon size	514	507
Average intron size	87	87
Median intron size	73	23
Intergenic size	15,484,048	15,422,613
Intragenic size	14,337,570	14,404,419
Number of introns in gene with largest splicing capacity	23	23
Number of putative secreted proteins	649	649
Number of predicted proteins with *e* value of *e* 5 or less	8157	8190
Genome read		
Number of reads	679.730.992	545.354.384
Number of read pairs	339.865.496	272.677.192
Number of bases	33.986.549.600	27.264.719.200

### Electrophoretic profile of *P. echinulatum*

**Fig. 1:**
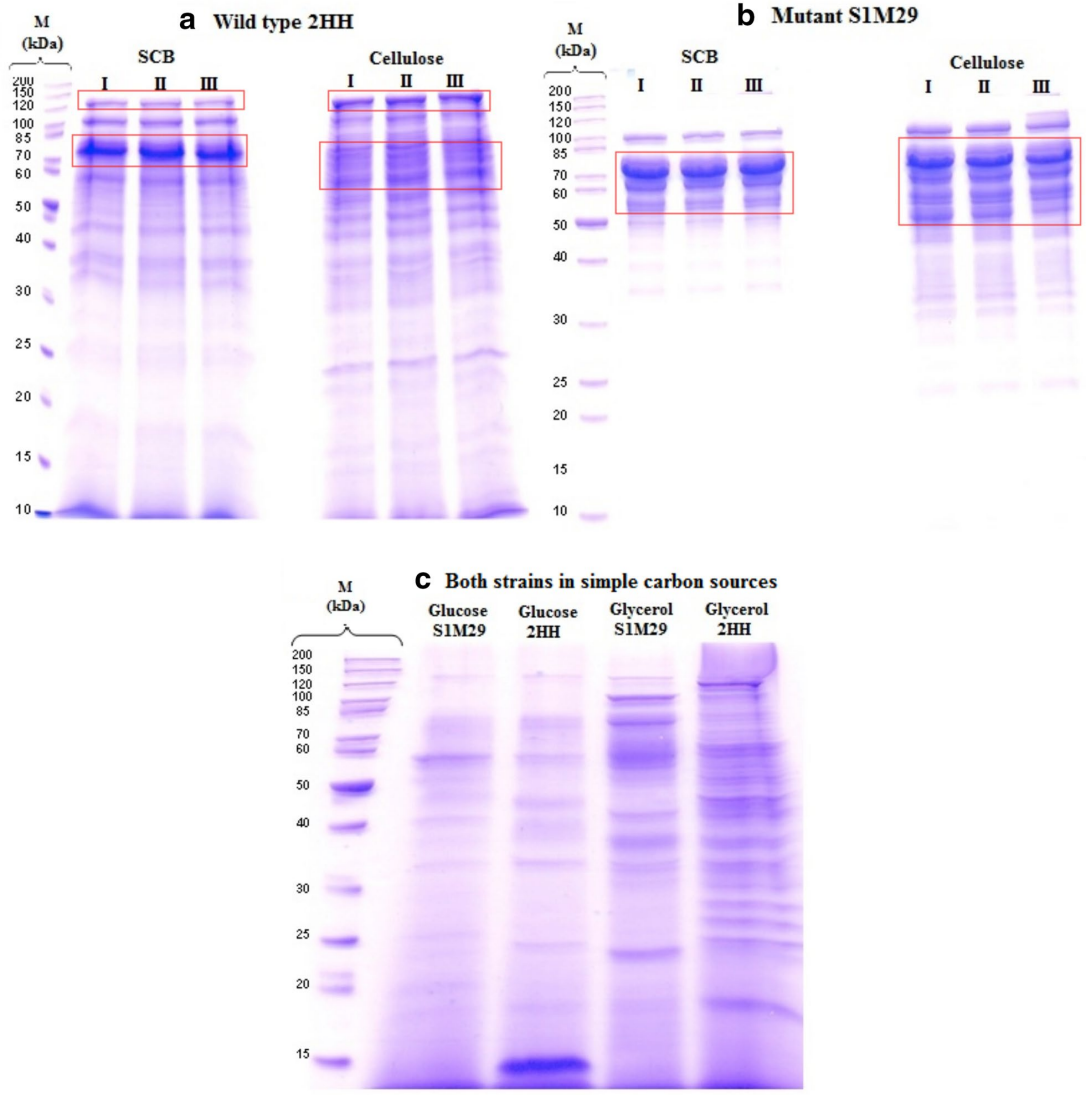
Electrophoretic profile of *Penicillium echinulatum* grown on SCB and cellulose at 96 h of cultivation. On the *left* **a**, 1-D PAGE of wild-type 2HH and on the *right* **b** 1-D PAGE of the mutant strain S1M29. I, II and III refer to triplicate. **c** Replicates for glucose and glycerol were performed, and profiles of wild-type 2HH and mutant S1M29 are shown intercalary in the gel above. *M* molecular marker, *kDa* molecular weight. *Red squares* indicate the differences in the level of some proteins that appear in greater quantity considering both strains and complex carbon sources

The analysis of the gels (1-D PAGE) of total proteins secreted by both strains of *P. echinulatum* grown on SCB, cellulose, glucose, and glycerol, after 96 h of culture, shows the diversity of molecular masses of proteins (Fig. 1). It is possible to see differences in the level of some proteins that appear in greater quantity in the mutant strain S1M29 (Fig. 1b—red squares) than in the wild-type 2HH when grown on cellulose or SCB, which is evidenced by proteomic data. Some of the proteins are correlated to cellulases and hemicellulases found in the range of 50–75 kDa (Tables 2, 3). On the other hand, protein bands of higher molecular weight (~120 kDa) are displayed only in the wild type (Fig. 1a—red squares).

**Table 2:** Identified cellulose-degradation/modifying enzymes and spectrum counts in different carbon sources by wild-type 2HH and mutant S1M29 of *Penicillium echinulatum* at 96 h during submerged cultivation

Accession number	Identified proteins	CAZyme	Organism	MW (kDa)	Secretion^b^	Spectral count^a^							
						2HH				S1M29			
						Glu	Gly	SCB	Cel	Glu	Gly	SCB	Cel
g2773	Cellobiohydrolase I	GH7 (CBM1)	*Penicillium decumbens*	56	Y	0	0	81	107	0	2	202	80
g8068	Cellobiohydrolase II	GH6 (CBM1)	*Penicillium decumbens*	49	Y	0	0	16	4	0	0	46	62
g8473	Endoglucanase 1	GH5 (CBM1)	*Penicillium echinulatum*	44	Y	0	0	19	27	0	1	75	59
g5809	Endoglucanase Cel5C	GH5 (CBM1, CBM46)	*Penicillium decumbens*	69	Y	0	1	28	30	0	1	56	58
g2788	Endoglucanase I	GH7 (CBM1)	*Penicillium decumbens*	50	Y	0	0	5	4	0	0	14	10
g2291	Endoglucanase II	GH5 (CBM1)	*Penicillium decumbens*	44	Y	0	0	4	4	0	0	13	7
g2659	Endo-β-1,4-glucanase	GH5 (CBM1)	*Penicillium oxalicum*	52	Y	27	0	0	3	49	1	0	6
g3996	Endo-β-1,4-glucanase	GH12	*Penicillium oxalicum*	26	Y	0	0	9	10	0	0	12	8
g7274	Endo-β-1,4-glucanase	GH5	*Penicillium oxalicum*	55	N	0	0	5	1	0	0	0	0
g6766	β-glucosidase I	GH3	*Penicillium decumbens*	93	Y	0	7	2	1	1	17	2	0
g8479	Related to β-glucosidase	GH132	*Colletotrichum gloeosporioides*	46	Y	0	2	0	22	4	0	0	0
g3303	Cellulose monooxygenase Cel61A	AA9	*Penicillium oxalicum*	26	Y	0	0	3	0	0	0	20	11
g8342	Cellulose monooxygenase	AA9	*Penicillium oxalicum*	45	Y	0	0	3	0	0	0	0	0
g5167	Swollenin	CBM1, CBM63	*Penicillium oxalicum*	52	Y	0	0	1	4	0	0	9	6
g1074	GMC oxidoreductase	AA3	*Neosartorya fischeri*	70	Y	0	0	0	0	0	1	0	0
g3947	GMC oxidoreductase	AA3	*Penicillium roqueforti*	67	N	0	4	0	0	0	0	0	0

^a^Secretomic analysis based on spectral counting. A quantitative analysis was conducted for samples grown on cellulose or SCB (mean of triplicates), while a semiquantitative analysis for samples grown on glucose or glycerol (one replicate) was performed. The complete protein reports are given in Additional file 3

^b^The secretion of each protein was verified by the softwares SignalP, SecretomeP, and YLoc. When at least two of three softwares give a positive result, the protein was considered secreted

**Table 3:** Identified hemicellulose-degradation enzymes and spectrum count in different carbon sources by wild-type 2HH and mutant S1M29 of *Penicillium echinulatum* at 96 h during submerged cultivation

Accession number	Identified proteins	CAZyme	Organism	MW (kDa)	Secretion^b^	Spectrum count^a^							
						2HH				S1M29			
						Glu	Gly	SCB	Cel	Glu	Gly	SCB	Cel
g2645	Xylanase	GH10 (CBM1)	*Penicillium decumbens*	44	Y	0	0	1	10	0	0	21	27
g1025	Endo-β-1,4-xylanase	GH10	*Penicillium oxalicum*	40	Y	0	0	12	21	0	0	0	1
g3831	Endo-β-1,4-xylanase	GH30	*Penicillium oxalicum*	53	Y	0	0	5	11	0	0	0	2
g5166	Endo-β-1,4-xylanase	GH11 (CBM1)	*Penicillium oxalicum*	31	Y	0	0	0	2	1	0	2	5
g3240	β-xylosidase	GH43	*Penicillium oxalicum*	64	Y	0	0	0	8	0	0	0	0
g1531	β-xylosidase	GH43	*Penicillium oxalicum*	38	Y	0	0	8	0	0	0	0	0
g2200	β-xylosidase	GH3	*Penicillium oxalicum*	85	Y	0	0	0	1	0	0	0	15
g5735	α-mannosidase	GH47	*Penicillium oxalicum*	56	Y	0	14	7	3	0	16	0	3
g395	Exo-α-1,6-mannosidase	GH125	*Sphaerulina musiva*	56	N	0	12	0	2	0	7	0	0
g5829	β-1,4-mannanase	GH5 (CBM1)	*Penicillium oxalicum*	47	Y	0	0	7	8	0	2	0	12
g8513	Sorbitol/xylulose reductase	GH99	*Aspergillus clavatus*	28	N	0	13	6	22	0	2	1	1
g315	Acetyl xylan esterase	CE1 (CBM1)	*Penicillium oxalicum*	41	Y	0	0	0	0	0	0	1	1
g2707	Acetyl xylan esterase	CE5	*Penicillium oxalicum*	24	Y	0	0	0	5	0	0	1	2

^a^Secretomic analysis based on spectral counting. A quantitative analysis was conducted for samples grown on cellulose or SCB (mean of triplicates), while a semiquantitative analysis for samples grown on glucose or glycerol (one replicate) was performed. The complete protein reports are given in Additional file 3

^b^The secretion of each protein was verified by the softwares SignalP, SecretomeP and YLoc. When at least two of three softwares give a positive result the protein was considered secreted

The analysis of electrophoretic profiles of both strains of *P. echinulatum* grown on glucose or glycerol (Fig. 1c) also reveals a significant number of bands with different molecular weights. However, the bands do not appear so intensely when compared with SCB or medium formulated with cellulose, which clearly indicates that the secretion of proteins changes according to the carbon source.

**Fig. 2:**
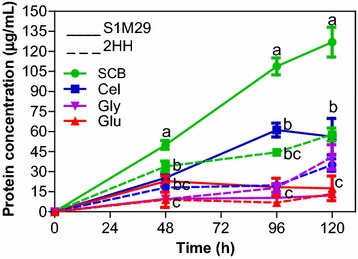
Protein concentration. Levels of protein concentration of *Penicillium echinulatum* wild-type 2HH and mutant S1M29 grown in four different carbon sources (SCB, cellulose, glucose, and glycerol) for 120 h. Protein concentration was performed according to the method of Bradford and is expressed in µg/mL

Figure 2 shows the protein concentration. The amount of proteins present in the media with cellulose or SCB after 48 h of cultivation is statistically higher for the mutant S1M29 compared to the wild-type 2HH. It is worth noting that the SCB medium presents the largest amount of protein. The amount of proteins secreted by the two strains in the media elaborated with glycerol or glucose slightly varied during the time course and corresponds to a smaller amount compared with cellulose and SCB. Still, the quantity of protein in the media formulated with glucose or glycerol did not differ statistically, even at 120 h of cultivation.

### Profiling of the *P. echinulatum* secretome for lignocellulose degradation

**Fig. 3:**
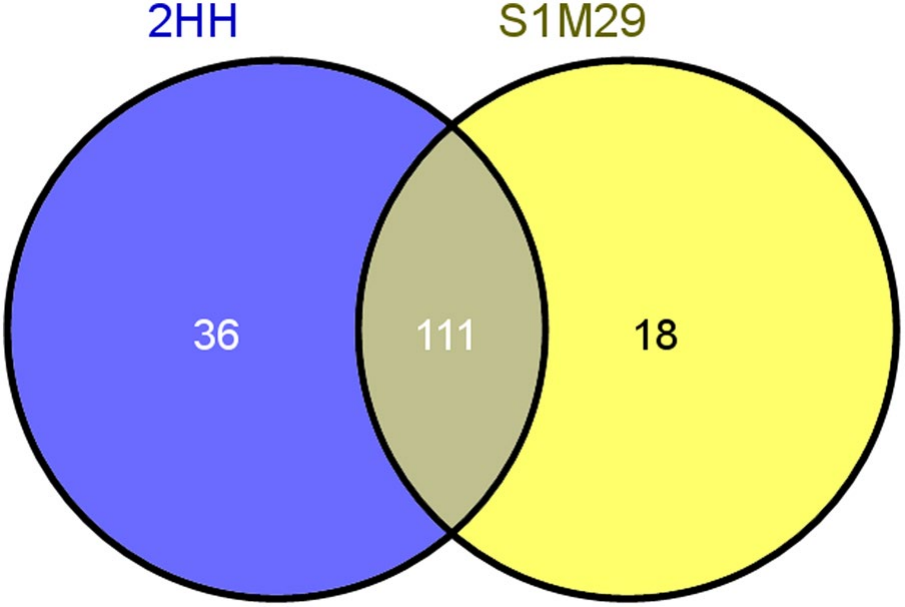
Venn diagram of secretome from *Penicillium echinulatum* strains. *Venn diagram* shows the shared secretome (*overlap*) and specific secretome for wild-type 2HH (*blue*) and mutant S1M29 (*yellow*)

The set of proteins found in secretome of both strains have a molecular weight in the range between 11 and 123 kDa. In the secretomic analyses of wild-type 2HH and mutant S1M29 from *P. echinulatum*, 165 proteins were found. In total, 36 proteins are found exclusively from the wild type, 18 proteins exclusively from the mutant, while 111 are commonly found in both strains (Fig. 3). Using bioinformatics tools to predict secreted proteins, it has been found that 117 proteins (71 %) of the identified proteins are predicted to contain signal peptide and/or are secreted by nonclassical routes.

**Fig. 4:**
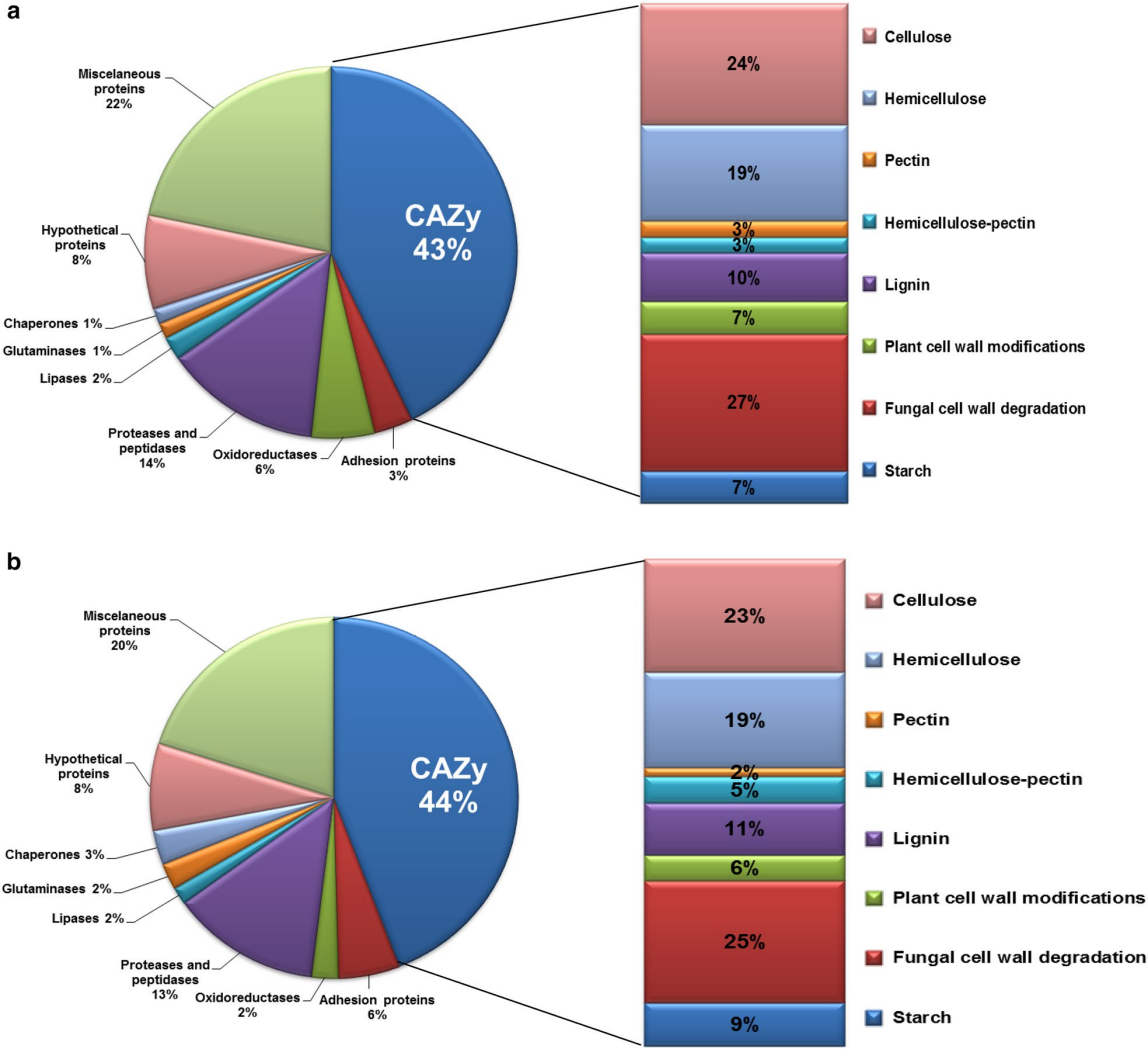
Distribution of proteins identified in secretome of *Penicillium echinulatum* strains. The proteins were classified according to the CAZy database. **a** The CAZy families identified in the wild type. **b** The CAZy families identified in the mutant

The main objective of this work was to find enzymes involved in the degradation of lignocellulosic biomass. Out of the 147 proteins found in the wild type, 63 proteins (~43 %) belonged to families described in the CAZy database [25]. Similarly, 57 proteins (~44 %) from 129 proteins found in the mutant belonged to families in the CAZy database (Fig. 4).

Among the proteins found in the secretomes of the wild-type and mutant, cellulases, hemicellulases, and fungal cell wall degradation enzymes were the majority enzymes, accounting for over 50 % of the identified proteins (Fig. 4). However, cellulases correspond to enzymes that were more expressed, according to the spectral counting analysis.

**Fig. 5:**
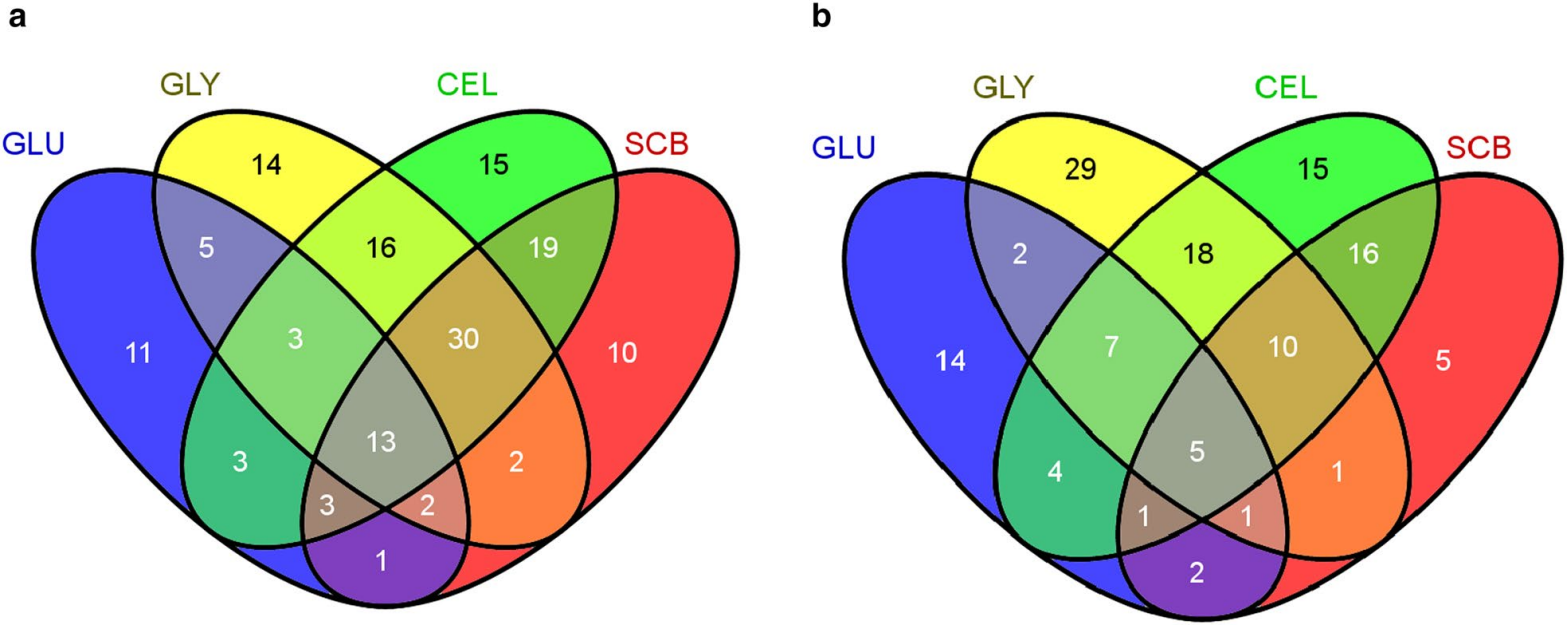
Venn diagram of secretomes from *Penicillium echinulatum* strains under each condition. **a** Distribution of 147 proteins identified in the secretome of wild-type 2HH, and **b** distribution of 129 proteins identified in the secretome of mutant S1M29, exclusive in each carbon source or shared between them. *GLU* glucose, *GLY* glycerol, *CEL* cellulose, and *SCB* sugar cane bagasse. The venn diagram was prepared by means of the tool, Venny (http://bioinfogp.cnb.csic.es/tools/venny/index.html)

Regarding the secretome profile found in wild-type 2HH of *P. echinulatum* the largest number of proteins were found in the medium formulated with cellulose (102 proteins, 15 exclusive), followed by glycerol (85 proteins, 14 exclusive) SCB (80 proteins, 10 exclusive) and glucose (41 proteins, 11 exclusive). From the 147 proteins found in the secretome of wild type, 13 were found in all carbon sources evaluated in this work (Fig. 5a).

In secretome profile of mutant strain S1M29 of *P. echinulatum*, the larger number of proteins was found again in the medium formulated with cellulose (76 proteins, 15 exclusive). Glycerol appears in the sequence with 73 proteins identified, and it has the greatest number of exclusive proteins (29). The medium formulated with SCB comes in third place (41 proteins, 5 exclusives), followed by glucose (36 proteins, 14 exclusives). Of the 129 proteins found in the secretome of the mutant strain, five were found in all four different culture media (Fig. 5b).

Among 36 proteins found only in the wild-type secretome, some have glycosyl hydrolase (GH) function, such as exo-β-1,3-glucanase (GH5), 1,3-β-glucanase (GH17), found exclusively in glucose, and chitinase (GH18), found only in glycerol (Table 10). In addition, endo-β-1,4-glucanase (GH5) (Table 2) and β-xylosidase (GH43) were found in cellulose or SCB (Table 3), and exo-α-l-1,5-arabinanase (GH93) was present only in cellulose (Table 4). Carbohydrate acetyl esterase (CE16) (Table 8) and cellulose monooxygenase (AA9) were also detected, and the latter secreted only in SCB (Table 2). As can be viewed in Additional file 1, some hypothetical and adhesion proteins, proteases, lipases, and other proteins were also found in the secretome of *P. echinulatum* wild type.

**Table 4:** Identified pectin-degradation enzymes and spectrum counts in different carbon sources by wild-type 2HH and mutant S1M29 of *Penicillium echinulatum* at 96 h during submerged cultivation

Accession number	Identified proteins	CAZyme	Organism	MW (kDa)	Secretion^b^	Spectrum count^a^							
						2HH				S1M29			
						Glu	Gly	SCB	Cel	Glu	Gly	SCB	Cel
g4880	Pectin lyase	PL1	*Penicillium oxalicum*	40	Y	0	0	0	1	12	0	0	0
g3609	Exo-α-l-1,5-arabinanase	GH93	*Penicillium oxalicum*	44	Y	0	0	0	2	0	0	0	0

^a^Secretomic analysis based on spectral counting. A quantitative analysis was conducted for samples grown on cellulose or SCB (mean of triplicates), while a semiquantitative analysis for samples grown on glucose or glycerol (one replicate) was performed. The complete protein reports are given in Additional file 3

^b^The secretion of each protein was verified by the softwares SignalP, SecretomeP and YLoc. When at least two of three softwares give a positive result the protein was considered secreted

Concerning the mutant strain secretome, 18 exclusive proteins were identified, among these also enzymes with GH function, such as α-l-arabinofuranosidase (GH62), found only in SCB and cellulose (Table 5) and β-1,6-glucanase (GH30) (Table 10). Esterases, such as acetyl xylan esterase (CE1) (Table 3) and enzymes related to the depolymerization of lignin (Table 6) were identified. Additional data also show the other exclusive proteins found refer to ribosomal, hypothetical, and adhesion proteins, lipases, and proteases/peptidases (Additional file 1).

**Table 5:** Identified hemicellulose/pectin-degradation enzymes and spectrum counts in different carbon sources by wild-type 2HH and mutant S1M29 of *Penicillium echinulatum* at 96 h during submerged cultivation

Accession number	Identified proteins	CAZyme	Organism	MW (kDa)	Secretion^b^	Spectrum count^a^							
						2HH				S1M29			
						Glu	Gly	SCB	Cel	Glu	Gly	SCB	Cel
g1097	α-l-arabinofuranosidase	GH62 (CBM1)	*Penicillium oxalicum*	41	Y	0	0	0	0	0	0	1	3
g7308	α-l-arabinofuranosidase	GH43	*Penicillium oxalicum*	34	Y	0	2	2	0	0	0	1	0
g1098	α-l-arabinofuranosidase	GH43 (CBM1)	*Penicillium oxalicum*	62	Y	0	0	0	1	0	0	0	1

^a^Secretomic analysis based on spectral counting. A quantitative analysis was conducted for samples grown on cellulose or SCB (mean of triplicates), while a semiquantitative analysis for samples grown on glucose or glycerol (one replicate) was performed. The complete protein reports are given in Additional file 3

^b^The secretion of each protein was verified by the softwares SignalP, SecretomeP and YLoc. When at least two of three softwares give a positive result the protein was considered secreted

**Table 6:** Identified lignin-degradation enzymes and spectrum counts in different carbon sources by wild-type 2HH and mutant S1M29 of *Penicillium echinulatum* at 96 h during submerged cultivation

Accession number	Identified proteins	CAZyme	Organism	MW (kDa)	Secretion^b^	Spectrum count^a^							
						2HH				S1M29			
						Glu	Gly	SCB	Cel	Glu	Gly	SCB	Cel
g1557	Isoamyl alcohol oxidase	AA7	*Talaromyces stipitatus*	66	Y	11	2	6	0	35	31	3	4
g1100	Isoamyl alcohol oxidase	AA7	*Arthroderma benhamiae*	58	Y	2	0	0	2	0	0	0	1
g5086	Isoamyl alcohol oxidase	AA7	*Aspergillus flavus*	67	Y	0	0	0	1	0	0	1	2
g6365	Related to Isoamyl alcohol oxidase	AA7	*Fusarium fujikuro*	69	Y	0	1	3	3	18	11	0	3
g7414	FAD dependent oxidoreductase	AA7	*Aspergillus clavatus*	54	Y	0	2	2	1	0	10	0	0
g5776	6-hydroxy-d-nicotine oxidase	AA7	*Aspergillus kawachii*	54	Y	3	4	2	2	5	6	0	2

^a^Secretomic analysis based on spectral counting. A quantitative analysis was conducted for samples grown on cellulose or SCB (mean of triplicates), while a semiquantitative analysis for samples grown on glucose or glycerol (one replicate) was performed. The complete protein reports are given in Additional file 3

^b^The secretion of each protein was verified by the softwares SignalP, SecretomeP and YLoc. When at least two of three softwares give a positive result the protein was considered secreted

Most of the proteins identified as exclusive in the secretome of the wild-type 2HH and the mutant strain S1M29 have a low number of spectral counting, which generates some analysis bias due to differences in the length of protein, the molecular weight, and ionization efficiency [26].

**Fig. 6:**
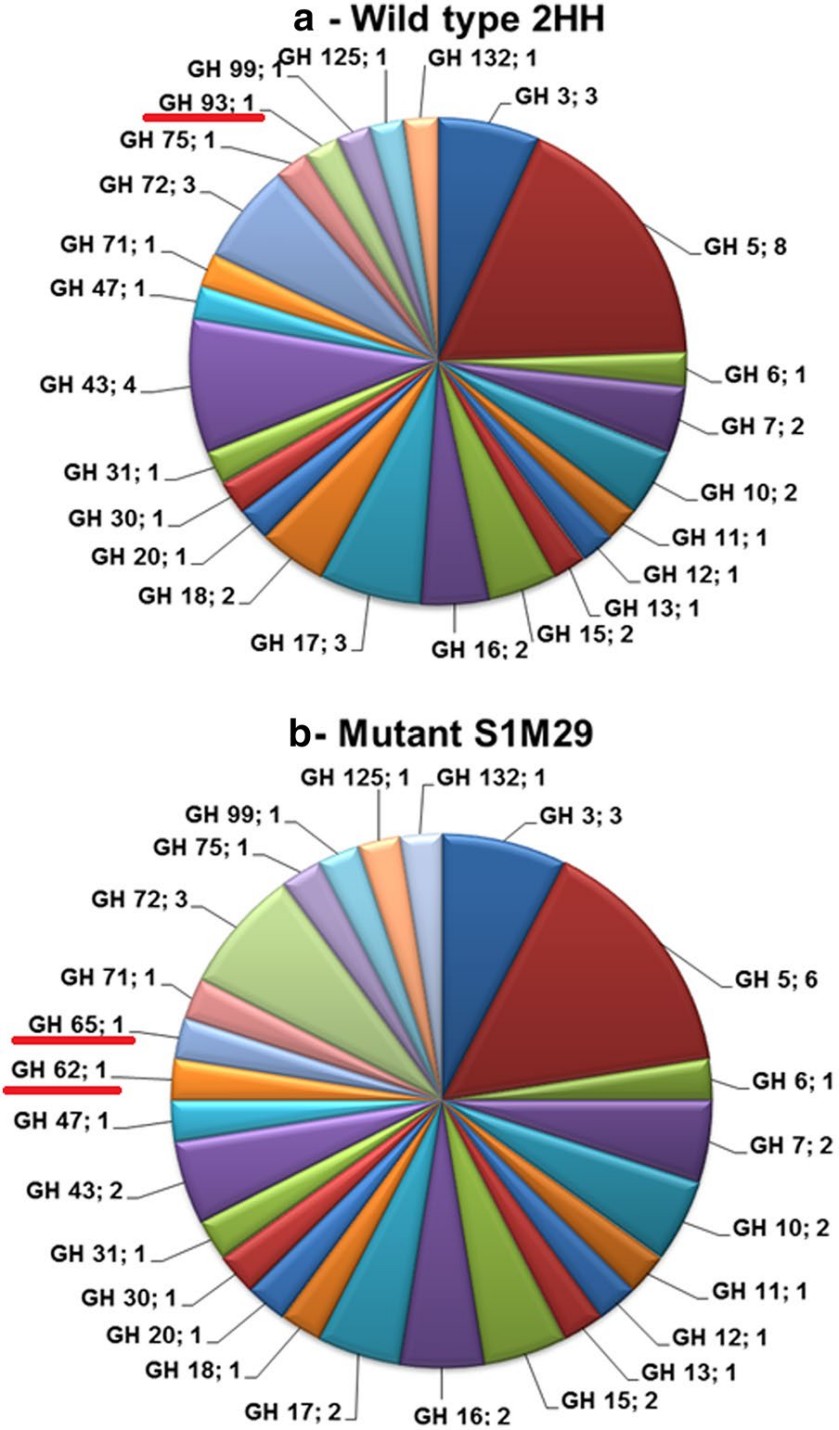
Distribution of GHs families in *Penicillium echinulatum* secretome. The proteins were classified according to CAZy database. **a** GHs identified in wild-type 2HH, **b** GHs identified in mutant S1M29, highlighting the presence of the families GH93 in 2HH (**a**) and GH62/GH65 in S1M29 (**b**). They are exclusive to each strain, although the number of spectra for this protein was low

Among the proteins belonging to the CAZy database in the wild-type 2HH, 45 proteins were found grouped in 24 different families of GHs (Fig. 6a), with the largest number of spectra belonging to cellobiohydrolases I and II (GH7 and GH6), endoglucanases (GH5 and GH7) and xylanase (GH10). These enzymes were basically identified only in the media formulated with SCB or cellulose. In the secretome profile of the mutant S1M29, 57 proteins belong to CAZy families, and once again, GHs were the most abundant (41 proteins grouped into 25 families), as can be seen in Fig. 6b.

Considering the CAZymes found in mutant and wild type, there were a greater number of spectra identified in the mutant, as can be seen especially in Tables 2 and 3. This happened with cellobiohydrolases I and II (GH7 and GH6), endoglucanases 1 and Cel5C, endoglucanase (GH5), and xylanase (GH10). In addition, these enzymes have been found with a higher number of spectra in SCB and cellulose. β-glucosidase I (GH3) was identified with a greater number of spectra in glycerol medium, with this number also larger in the mutant strain (Table 2). However, another protein related to β-glucosidase was found in the wild type with a considerable amount of spectra in cellulose.

The number of spectra for exo-β-1,3-glucanase (GH17) in both strains cultivated in glucose is relatively larger than has been identified in other carbon sources, especially in the wild type (Table 10), and is related to the degradation of the fungal cell wall. Other GHs found in the wild type with larger spectra in glucose and glycerol than in SCB and cellulose, which are also related to the degradation of the fungal cell wall (Table 10), were chitin glucanosyltransferase (GH16), chitosanase (GH75) and β-*N*-acetylhexosaminidase (GH3). As shown in additional file the presence of lysophospholipase phospholipase B (Additional file 1) with a greater number of spectra in the wild type, especially in medium prepared with glucose or glycerol, is a further indication of the arsenal of enzymes produced by the wild type for the degradation of fungal cell wall.

Other enzymes with GH function found in both strains with a reduced number of spectra were putative endoxylanase (GH30), α-mannosidase (GH47), with the largest number of spectra in glycerol; β-1,4-mannanase (GH5), found in SCB and cellulose; β-xylosidase (GH3), found only in the media formulated with cellulose, in both strains (Table 3). Distinct endoglucanases (GH5 and GH12) were expressed differently in the culture media—GH5 with more spectra in glucose, but GH12 in SCB and cellulose (Table 2).

The presence of enzymes related to starch degradation were also found in both strains, although with a lower number of spectra, as the case of α-amylase (GH13), α-glucosidase (GH31) and two glucoamylases (GH15), the latter being identified only when glucose or glycerol was used as a carbon source (Table 7).

**Table 7:** Identified starch-degradation enzymes and spectrum counts in different carbon sources by wild-type 2HH and mutant S1M29 of *Penicillium echinulatum* at 96 h during submerged cultivation

Accession number	Identified proteins	CAZyme	Organism	MW (kDa)	Secretion^b^	Spectrum count^a^							
						2HH				S1M29			
						Glu	Gly	SCB	Cel	Glu	Gly	SCB	Cel
g4201	α-amylase	GH13	*Penicillium oxalicum*	74	Y	0	2	2	1	0	1	0	6
g5478	Glucoamylase Amy15A	GH15 (CBM20)	*Penicillium oxalicum*	66	Y	1	1	0	0	9	10	0	0
g36	Glucoamylase	GH15 (CBM20)	*Penicillium oxalicum*	68	Y	1	0	0	0	14	0	0	0
g7292	α-glucosidase	GH31	*Penicillium oxalicum*	108	Y	0	7	0	0	0	1	0	0
g1651	α-trehalase	GH65	*Penicillium oxalicum*	119	Y	0	0	0	0	0	23	0	0

^a^Secretomic analysis based on spectral counting. A quantitative analysis was conducted for samples grown on cellulose or SCB (mean of triplicates), while a semiquantitative analysis for samples grown on glucose or glycerol (one replicate) was performed. The complete protein reports are given in Additional file 3

^b^The secretion of each protein was verified by the softwares SignalP, SecretomeP and YLoc. When at least two of three softwares give a positive result the protein was considered secreted

Six carbohydrate esterases were identified in both strains, with a reduced number of spectra, grouped in four different families, including carboxylesterases (CE10), homoserine acetyltransferase (CE1), carbohydrate acetylesterase (CE16) (Table 8) and acetyl xylan esterase (CE1 and CE5) (Table 3).

**Table 8:** Identified enzymes involved in plant cell wall modifications and spectrum counts in different carbon sources by wild-type 2HH and mutant S1M29 of *Penicillium echinulatum* at 96 h during submerged cultivation

Accession number	Identified proteins	CAZyme	Organism	MW (kDa)	Secretion^b^	Spectrum count^a^							
						2HH				S1M29			
						Glu	Gly	SCB	Cel	Glu	Gly	SCB	Cel
g1590	Carboxylesterase, type B	CE10	*Penicillium roqueforti*	62	Y	9	1	0	2	0	1	0	5
g7004	Carbohydrate acetyl esterase	CE16	*Penicillium oxalicum*	33	Y	0	0	0	5	0	0	0	0
g5807	Homoserine acetyltransferase	CE1	*Talaromyces marneffei*	37	N	0	0	1	1	0	0	0	0
g1708	Carboxylesterase, type B	CE10	*Penicillium roqueforti*	61	Y	0	0	0	1	0	0	0	1

^a^Secretomic analysis based on spectral counting. A quantitative analysis was conducted for samples grown on cellulose or SCB (mean of triplicates), while a semiquantitative analysis for samples grown on glucose or glycerol (one replicate) was performed. The complete protein reports are given in Additional file 3

^b^The secretion of each protein was verified by the softwares SignalP, SecretomeP and YLoc. When at least two of three softwares give a positive result the protein was considered secreted

The same families of auxiliary redox enzymes (AA9), which act together with CAZymes, were found in both strains. Cellulose monooxygenase Cel 61A (AA9/g3303) was one of the most produced enzymes when we consider S1M29 in the medium prepared with SCB and cellulose. For 2HH, the presence of this AA9 is observed only in the medium with SCB, although the presence of another AA9 (putative) is possible in the SCB medium.

The presence of only one polysaccharide lyase, the pectin lyase (PL1) and one exo-arabinanase was verified (Table 4). Arabinofuranosidase was also found in less quantity (Table 4).

The secretome analysis has shown that approximately 30 % of the identified CAZymes have carbohydrate-binding module (CBMs). Eight different CBM families were identified, with the CBM1 as the most abundant. In the case of endoglucanase Cel5C (GH5/g5809), two CBMs were found attached to the same CAZyme: CBM46 and CBM1, and also two CBMs for swollenins: CBM63 and CBM1 (Table 2).

Additional data features the presence of xylose reductase in both strains grown in SCB and cellulose, and they may indicate the potential of these broths for enzymatic production of xylitol and perhaps the potential use as a facilitator for xylose-to-ethanol conversion by other microorganisms (refer to Additional file 1). Xylulose reductase was also found (Table 3), but this enzyme does not participate in the conversion of xylose to xylulose for ethanol production [27].

Some enzymes involved in the degradation of lignin were detected. In the secretome of mutant strain, isoamyl alcohol oxidase (AA7) was found in a large number of spectra in glucose and glycerol (Table 6). GMC oxidoreductase (AA3), an enzyme which acts both in cellulose and lignin—possibly related to cellobiose dehydrogenase [28, 29]—was found in the medium with glycerol (Table 2). Manganese superoxide dismutase and glutathione *S*-transferase were also identified, as shown in Additional file 1.

In Table 9, the main differences between the secretomes of the wild type and mutant are represented, regarding the identification of key proteins in the lignocellulosic biomass degradation. For most enzymes analyzed, there are statistical differences (*t* test *p* < 0.05) between the number of spectra found in SCB or cellulose, when comparing the spectra in wild type with the mutant. That difference is clearly shown, i.e., the expression of these enzymes in SCB by the mutant. The expression of cellobiohydrolases, endoglucanases, and xylanase reveal the mutant S1M29 greater potential for biomass hydrolysis.

**Table 9:** Key proteins involved in the lignocellulosic biomass degradation and its significant difference between the spectra produced in SCB and cellulose

Accession number	Identified proteins	CAZyme	Spectrum count								*T* test^a^ (*p* < 0.05)	*T* test^a^ (*p* < 0.05)
			2HH				S1M29					
			Glu	Gly	SCB	Cel	Glu	Gly	SCB	Cel	SCB	CEL
g2773	Cellobiohydrolase I	GH7 (CBM1)	0	0	81	107	0	2	202	80	*0.019*	0.29
g8068	Cellobiohydrolase II	GH6 (CBM1)	0	0	16	4	0	0	46	62	*0.027*	*0.0045*
g5809	Endoglucanase Cel5C	GH5 (CBM1, CBM46)	0	1	28	30	0	1	56	58	*0.0018*	*0.0032*
g2788	Endoglucanase I	GH7 (CBM1)	0	0	5	4	0	0	14	10	*0.0011*	0.10
g2291	Endoglucanase II	GH5 (CBM1)	0	0	4	4	0	0	13	7	*0.0073*	*0.0072*
g3996	Endo-β-1,4-glucanase	GH12	0	0	9	10	0	0	12	8	*0.045*	0.064
g3303	Cellulose monooxygenase Cel61A	AA9	0	0	3	0	0	0	20	11	*0.0013*	*0.00060*
g5167	Swollenin	CBM1, CBM63	0	0	1	4	0	0	9	6	*0.00030*	0.23
g2645	Xylanase	GH10 (CBM1)	0	0	1	10	0	0	21	27	*0.00010*	*0.012*
g1025	Endo-β-1,4-xylanase	GH10	0	0	12	21	0	0	0	1	0.0056	*0.038*
g1531	β-xylosidase	GH43	0	0	8	0	0	0	0	0	*0.0020*	1.00
g5829	β-1,4-mannanase	GH5 (CBM1)	0	0	7	8	0	2	0	12	*0.0046*	0.42
g5309	Exo-β-1,3-glucanase	GH5	0	3	5	8	0	0	0	0	*0.00010*	*0.010*

^a^ Statistical approach (*T* test, *p* < 0.05) was performed between the strains for SCB and cellulose media. Proteins with statistically significant differences are highlighted in italics

When comparing the total number of spectra found in SCB or cellulose between strains, it is found that 77 % of the spectra found in SCB in the mutant strain correspond to enzymes relating to cellulose and hemicellulose degradation, whereas in the medium with cellulose, 58 % of these spectra are found. In contrast, when the same comparison is made with the wild type, there were a greater number of spectra (~38 %) in cellulose compared to SCB (~31 %). It is suggested that SCB has become a better inducer of cellulolytic and hemicellulolytic enzymes, at least for the mutant strain. Considering either glucose or glycerol as carbon source, the amounts of cellulases and hemicellulases produced corresponded to less than 10 % of the total spectra for both strains.

Taking another approach, Additional file 2 demonstrates that, considering the total number of spectra, when we look at the ten most expressed proteins in each carbon source for each strain, it was again evident that the secretome of mutant strain in the media with SCB or cellulose is more geared for the production of cellulolytic and hemicellulolytic enzymes than the wild type; more than 70 % of these proteins correspond to cellulases and hemicellulases. Meanwhile, the secretome of media containing glucose or glycerol have a protein complex consisting of a majority of proteins related to degradation of fungal cell wall, adhesion proteins, and proteases; only two cellulases (g2659/g6766) and one hemicellulase (g5735) were identified among the ten most expressed proteins in these conditions and only in the mutant strain.

### Enzymatic analysis of the *P. echinulatum* secretome

**Fig. 7:**
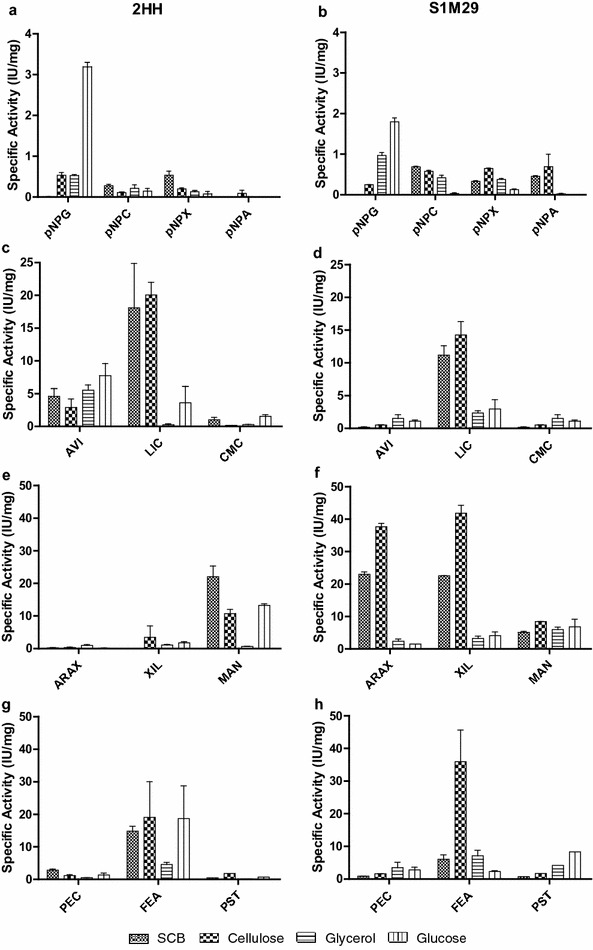
Specific enzymatic activity of *Penicillium echinulatum* strains in submerged cultures using different carbon sources at 96 h of cultivation. The *graphs* on the *left* correspond to the wild-type 2HH, and the *graphs* on the *right* correspond to the mutant strain S1M29. The hydrolytic potential of the broths of *Penicillium echinulatum* strains was tested on different substrates. **a**, **b** β-glucosidases. **c**, **d** endoglucanases. **e**, **f** xylanase, and **g**, **h** pectinase. pNPG *p*-nitrophenyl-β-d-glucopyranoside, pNPC *p*-nitrophenyl-β-d-cellobioside, pNPX *p*-nitrophenyl-β-d-xylopyranoside, pNPA *p*-nitrophenyl-α-d-arabinofuranoside, *AVI* Avicel^®^, *LIC* Lichenan, *CMC* carboxymethyl cellulose, *ARAX* rye arabinoxylan, *XIL* xylan, *MAN* mannan, *PEC* pectin, *FEA* feruloyl acetate, *PST* potato starch

For further validation of proteomic data, the hydrolytic potential of stocks of the wild-type 2HH and mutant S1M29 strains was assessed using nine different polysaccharide substrates and four *p*-nitrophenyl (pNP) substrates. Some results in Fig. 7 are shown in another work, in the form of absolute enzymatic activity (unpublished data). However, due to proteomic analysis, some parts of these data are presented here in the form of specific enzymatic activity.

The highest activity of β-glucosidases (Fig. 7a,b), using the substrate *p*-nitrophenyl-β-d-glucopyranoside (pNPG), was found for both strains, in the media elaborated with glucose or glycerol, which is according to the proteomic data. The largest number of spectra for cellobiohydrolases, dosed with *p*-nitrophenyl-β-d-cellobioside (pNPC) (Fig. 7b), was found in the media formulated with SCB or cellulose, mainly in mutant broths (Table 2).

Regarding endoglucanases activities, when the substrate lichenan has been employed, the specific activity was higher in media prepared with cellulose or SCB, for both strains, which agrees with proteomics data (Fig. 7c, d). However, upon checking the endoglucanases found in the *Penicillium* secretome, some spectra were found in glycerol and glucose media, for both strains (Table 2, protein g2659). It would explain the higher activities of endoglucanases in glycerol and glucose media, when the substrate carboxymethyl cellulose (CMC) was employed (Fig. 7c, d). The higher enzymatic activities on Avicel^®^ by 2HH may be explained due the presence of multienzymatic complexes (data not shown) or higher diversity of proteins bands that can contribute to degradation of microcristaline cellulose (Fig. 7c, d).

The specific activity of xylanase, dosed with xylan from beechwood substrate (Fig. 7e, f), was higher in the medium formulated with cellulose or SCB, especially for mutant strains, which stood out with respect to the wild type, as can be seen in Table 3. The presence of β-xylosidases found in both strains with more spectra in cellulose and SCB also showed specific activities in these media (Fig. 7a, b). Higher specific activities of hemicellulases in media prepared with cellulose can be explained by the characterization of carbon sources employed. The cellulose used in this work consists of 12.19 % xylose, while SCB employed consists of 6.57 % xylose [30].

Pectinase activity was low, which is consistent with a proteome composed predominantly by cellulases (Fig. 7g, h). The low arabinofuranosidase activities, dosed with pNPA (Fig. 7a, b), in both strains are in accord with the low expression of this enzyme in the proteomic analysis. The absence of arabinose in media prepared with cellulose and the low amount of this sugar in the media prepared with SCB (~2 %) corroborate these results.

Different esterases were found in both strains, more spectra in cellulose, such as CE1, CE5, and CE10. The highest specific activity for both strains was also displayed in the medium elaborated with cellulose (Fig. 7g, h). The specific activity in starch from potato was also higher when the mutant strain was used. In both strains, the highest activity was detected in the medium prepared with glucose, followed by the one made with glycerol. However, there were limitations to establishing some relationships between enzyme activities with the results obtained in secretomic broths. This is possibly due to synergistic interactions between enzymes.

## Discussion

Microbial cellulase and hemicellulase production is dependent on the carbon source [31]. The substrates used in this study have provided a high heterogeneity of secreted proteins due to the substrate and the protein profile related to each strain. The main objective of this work was achieved; that is, proteins and enzymes related to degradation of biomass, in particular the CAZymes, were found in *P. echinulatum* and the enzyme control change according to the different carbon source. Also, it is possible to confirm that both strains have the potential for hydrolysis of the cell wall, highlighting the specificities of the mutant strain.

Some proteomic studies involving the more efficient producer of cellulases known, i.e., *T. reesei*, have also been performed [32–35] and some results of these studies can be comparable to *P. echinulatum*. For example, in these studies involving the wild-type QM6a and the mutant RUT-C30 of *T. reesei* grown in media containing cellulose, corn straw, and sawdust, the authors concluded that lignocellulolytic enzyme in the secretome of both strains of *T. reesei* are dependent of lignocellulosic carbon sources [32]. Moreover, the functional classification of these biological quantified proteins revealed that 31.3 and 17.9 % correspond to cellulases and hemicellulases, respectively, and similar data with the amount of these enzymes, especially hemicellulases, in *P. echinulatum* secretome (Fig. 4).

Another similar study was conducted with *Trichoderma harzianum* grown on glucose, carboxymethyl cellulose, xylan, and sugar cane bagasse [33]. The characterization of *T. harzianum* secretome revealed that sugar cane bagasse induced greater cellulolytic and xylanolytic activities compared to the other substrates. The secretome analysis identified a wide range of proteins, including CAZymes. Although the secretome induced by sugar cane bagasse has the greatest cellulolytic and xylanolytic activities, it does not correspond to a higher complexity of proteins, since the induced secretome by carboxymethyl cellulose was significantly more diverse.

For *P. echinulatum*, sugar cane bagasse also induced a greater diversity of proteins being cultivated with the mutant strain, while cellulose was best for the wild type. As can be seen in Table 2, the highest amount of cellulases enzymes spectra were even checked in the sugar cane bagasse medium, suggesting that the genes related to degradation of lignocellulosic biomass are potentially transcribed during the cultivation of the fungus, but the relative proportions of the expressed proteins may vary widely depending on the growth medium and the cultivation conditions [34]. For instance, the extracellular cellulolytic system of *T. reesei* in response to 1 mmol/L sophorose is composed of 70 % cellobiohydrolases, 30 % endoglucanases and only 1 % of β-glucosidase [35]. In our study, however, when *P. echinulatum* grows in sugar cane bagasse, which proved to be ideal for production of cellulases by the mutant strain, the cellulolytic complex consists of 55 % cellobiohydrolases, 38 % endoglucanases, and 1 % β-glucosidases, being that the mutant secretes approximately two to three times more cellulases compared to the wild type, as has also been suggested for *T. reesei* [34].

These data lead to note that *P. echinulatum* presents a cellulolytic complex similar to *T. reesei*. However, as reported by Martins et al. [36], *P. echinulatum* shows higher relations between β-glucosidases and filter paper activity than *T. reesei*. This can be seen when glucose or glycerol are used as carbon sources for β-glucosidases production (Table 2 and Fig. 7a, b).

The genus *Aspergillus* is also reported in the literature as a good producer of enzymes related to degradation of biomass, and the secretome of different species has been studied [37–39]. A time course analysis of the extracellular proteome of *Aspergillus nidulans* growing on sorghum stover was also evaluated [38], finding several proteins in the secretome identified in *P. echinulatum* and additionally, mainly hemicellulases, followed by cellulases, polygalacturonases, chitinases, esterases, and lipases. Other important enzymes to reduce the recalcitrances of the biomass to hydrolysis, cellobiose dehydrogenase, and feruloyl esterase were found in great abundance. *Aspergillusniger* is also reported as an excellent producer of pectinases [39]. Although pectinases have been identified in *P. echinulatum* secretome, their diversity and amount are considerably smaller in comparison with other lignocellulolytic enzymes found.

*Penicillium* species are among many filamentous fungi whose secretome has been described in the last 10 years. Compared with *T. reesei*, lignocellulolytic enzyme systems produced by many *Penicillium* species have generally better performances (higher cellulose conversion at equal protein loadings) in lignocellulose hydrolysis [12]. Interestingly, *P. decumbens* [23] secretome showed a more diversified system of lignocellulolytic enzymes than *T. reesei*, particularly for cellulose binding domain-containing proteins and hemicellulases. Moreover, the proteomic analysis of *P. decumbens* revealed that the production of lignocellulolytic enzymes is greater in media containing cellulose and wheat bran than in glucose, which agrees with part of our studies.

Liao et al. [13] verified that a complex substrate composed of cellulose and xylan, simulated an artificially plant biomass, is more efficient than purified cellulose at inducing lignocellulolytic enzyme production in *Penicillium oxalicum* GZ-2. The addition of xylan to the cellulose culture did not affect fungal growth but significantly increased the activities of cellulase and hemicellulase. However, in *T. reesei* RUT-C30, cellulose induces a more efficient response.

Relating these results with *P. echinulatum,* we can conclude that the same relationship exists between *P. echinulatum* S1M29 and *P. oxalicum* GZ-2, at least, when we compare the numbers of cellulase and hemicellulase spectra and the protein concentrations in SCB. The data indicate that the mutant S1M29 is more suitable for the production of enzymes to hydrolyze the plant cell wall in comparison with the wild type, especially when it was grown in a culture medium formulated with SCB. This information is relevant for the production of enzyme complexes at lower cost, since there is a greater amount of proteins secreted by the mutant to degrade lignocellulosic wastes that are less costly and more complex.

In the production of second-generation ethanol, the cost of the enzyme is one of the major bottlenecks, and the cost of the substrate for the enzyme production can contribute decisively to this value; a ton of cellulose used for the cellulases production costs around US$1000 per ton, while the steam explosion SCB, is commercialized at US$40 a ton.

However, when we look at the specific activities*, P. echinulatum* S1M29 has the same behavior as *T. reesei* RUT-C30, where cellulose induces better enzymatic activities, at least for hemicellulose (Fig. 7f). This can be explained by the fact that the cellulose used in this work has more xylose than the SCB. Moreover, SCB used in our study was pretreated by steam explosion, and this process is characterized by the removal of a part of hemicellulose [40]; because of this, the lowest concentration of xylose in SCB did not favor the increased production of xylanase.

When *P. oxalicum* was grown in a medium with glycerol or glucose as the sole carbon source, low activities of cellulase and xylanase were detected, suggesting that some components are expressed at basal levels. When lignocellulosic substrates (e.g., cellulose, xylan, xylose, and wheat bran) were used as carbon sources, significantly higher lignocellulolytic enzyme activities were detected. The repressing effect of glucose and inductive effect of cellulose on the expressions of cellulases and xylanases were verified at gene-transcriptional levels [12]. These statements are consistent with the roles of each component in the production of lignocellulolytic enzymes by *P. echinulatum,* verified in our research.

Few data related to genetics of *P. echinulatum* have been published until now. Rubini et al. [41] isolated, cloned, and expressed one endoglucanase *egl 1* of the mutant *P. echinulatum* 9A02S1, and found high hydrolytic activity in a medium formulated with CMC. Endoglucanasic activity is a feature of *P. echinulatum* that remained in S1M29 strain and is according to the cellulolytic activities detected in this strain, as can be seen in Table 2 (protein g8473), showing that it is well expressed.

The use of different carbon sources for the production of cellulases and hemicellulases by *P. echinulatum* was previously reported by Novello et al. [21] and Schneider et al. [22]. Novello et al. [21] studied the production of endoglucanases, β-glucosidases, and xylanases by strains 2HH, 9A02S1, and S1M29 of *P. echinulatum*, employing cellulose, glucose, and xylose as carbon sources and reported that xylose acts as an inducer for the production of xylanases and cellulases, in particular, endoglucanases. The authors also found higher enzyme titers in cultures carried out with mutant strains 9A02S1 and S1M29 than those with the wild-type 2HH.

Schneider et al. [22] studied the effect of six different carbon sources (sucrose, glucose, glycerol, cellulose *Celuflok*^®^, untreated elephant grass, and SCB) in the production of enzymes by the mutant strain S1M29 of *P. echinulatum*. Among the six carbon sources, cellulose and SCB were the most suitable for the production of filter paper activity, endoglucanase, xylanases, and β-glucosidases. However, sucrose and glucose showed β-glucosidase activities similar to those obtained with the insoluble sources. The presence of these enzymes, especially β-glucosidase, in the work of Schneider et al. [22], with more activity at 96 h (at the end of the cultivation process), justifies the choice of 96-h duration for proteome analysis in the present study.

Some enzymes detected, such as β-1,3-glucanase (GH17, Table 10), do not have a secretion signal and may be related to a possible indication of cell wall autolysis at 96 h of cultivation, as reported by Vaheri et al. [42] and Fontaine et al. [43]. Many other fungal cell wall degrading enzymes, such as chitinases (GH18, Table 10), exo-β-1,3-glucanase (GH17, Table 10), and others already cited (chitosanase, β-*N*-acetylhexosaminidase, and lysophospholipase), may also be related to cell lysis. All these enzymes can be induced simply by the same sugars as the other CAZymes. This may be an indication that 2HH responds worse to stress than S1M29, and thus, produces a smaller amount of enzymes, which actually contributes to the degradation of biomass. It is suggested that the two strains deal with stress differently; the mutant is at a less-advanced stage of stress using mainly oxidative enzymes to maintain itself, while the wild type has passed through this stage and is already in a degrading process. From the analysis of the mycelial growth on glucose (data not shown), higher concentration of biomass has been found in wild type in relation to mutant up to 96 h of cultivation. Therefore, the greater growth of wild type on carbon sources of facilitated uptake, such as glucose, may be providing the production of these enzymes related to degradation of fungal cell walls, whereas the mutant makes use of more complex carbon sources for the production of enzymes related to degrading biomass.

**Table 10:** Identified fungal cell wall degradation/modifying enzymes and spectrum counts in different carbon sources by wild-type 2HH and mutant S1M29 of *Penicillium echinulatum* at 96 h during submerged cultivation

Accession number	Identified proteins	CAZyme	Organism	MW (kDa)	Secretion^b^	Spectrum count^a^							
						2HH				S1M29			
						Glu	Gly	SCB	Cel	Glu	Gly	SCB	Cel
g7097	Chitin glucanosyltransferase	GH16	*Penicillium oxalicum*	39	Y	41	11	12	13	32	0	9	10
g1080	Chitinase1	GH18	*Penicillium oxalicum*	49	Y	1	25	0	1	0	105	0	1
g8496	Chitinase 1, class 5	CBM50	*Grosmannia clavigera*	37	Y	16	0	0	0	0	0	0	0
g1258	Exo-β-1,3-glucanase	GH17	*Penicillium oxalicum*	47	Y	72	5	4	4	15	10	1	3
g4052	β-1,3-glucanosyltransferase	GH17	*Penicillium oxalicum*	33	Y	9	8	3	15	4	9	1	4
g284	β-*N*-acetylhexosaminidase	GH3	*Penicillium oxalicum*	38	Y	29	19	7	3	0	10	0	0
g244	β-1,3-glucanosyltransglycosylase	GH72	*Penicillium oxalicum*	49	Y	0	9	7	6	0	11	1	6
g3495	Chitosanase	GH75	*Penicillium oxalicum*	37	Y	30	12	3	3	1	2	0	2
g5309	Exo-β-1,3-glucanase	GH5	*Penicillium oxalicum*	45	Y	0	3	5	8	0	0	0	0
g6946	Chitin glucanosyltransferase	GH16 (CBM 18)	*Penicillium oxalicum*	47	Y	5	6	5	2	0	3	1	2
g980	β-1,6-*N*-acetyl glucosaminidase	GH20	*Penicillium oxalicum*	67	Y	0	4	4	2	0	4	0	1
g2799	β-1,3-glucanosyl transglycosylase	GH72	*Penicillium oxalicum*	53	Y	0	0	2	1	0	0	0	1
g1191	β-1,3-glucanosyl transglycosylase	GH72 (CBM 43)	*Penicillium oxalicum*	57	Y	2	0	1	0	0	0	0	1
g3950	Exo-β-1,3-glucanase	GH5	*Penicillium oxalicum*	46	Y	0	1	0	0	0	3	0	0
g6368	Chitinase	GH18	*Penicillium oxalicum*	47	Y	0	3	0	3	0	0	0	0
g4641	β-1,3-glucanase	GH17	*Penicillium oxalicum*	68	N	3	3	0	0	0	0	0	0
g5953	β-1,6-glucanase	GH30	*Penicillium oxalicum*	52	Y	0	0	0	0	0	0	1	0
g8051	α-1,3-glucanase	GH71 (CBM24)	*Penicillium oxalicum*	114	Y	7	0	0	2	0	0	0	5

^a^Secretomic analysis based on spectral counting. A quantitative analysis was conducted for samples grown on cellulose or SCB (mean of triplicates), while a semiquantitative analysis for samples grown on glucose or glycerol (one replicate) was performed. The complete protein reports are given in Additional file 3

^b^The secretion of each protein was verified by the softwares SignalP, SecretomeP and YLoc. When at least two of three softwares give a positive result the protein was considered secreted

The only secretome work on *P. echinulatum* done so far was conducted by Ribeiro et al. [5], employing the intermediate mutant 9A02S1. In their study, the secretome profiles of *P. echinulatum* after growing on integral sugar cane bagasse, microcrystalline cellulose, and three types of pretreated sugar cane bagasse were evaluated using shotgun proteomics. The same study revealed that the enzymatic repertoire of *P. echinulatum* is geared mainly toward producing enzymes from the cellulose complex (endoglucanases, cellobiohydrolases, and β-glucosidases). Glycoside hydrolase family members, important in terms of biomass-to-biofuel-conversion strategies, were identified—including endoglucanases GH5, 6, 7, 12, and 17; β-glycosidase GH3; xylanases GH10 and GH11, as well as debranching hemicellulases from GH43, GH62, and CE2; and pectinases from GH28.

The above results as found in that study agree with the information obtained from the present study, conducted with 2HH and S1M29 strains, where a larger amount of spectra in most enzymes was verified in media formulated with cellulose or SCB (in our study, pretreated by steam explosion), relating to degradation of biomass, such as cellobiohydrolases I and II (GH 6 and 7), endoglucanases 1 and Cel5C (GH5), and xylanases (GH10), demonstrating the potential of producing these enzymes especially by mutant strain S1M29. Although there is not much difference in enzyme diversity between the wild type and the mutants, the mutant S1M29 secretes greater diversity of glycosyl hydrolases compared to the mutant 9A02S1, e.g., GH15, 16, 30, 31, 72, 75, 99, 125, and 132, as well as other CAZy enzymes (Table 11). The specific activity also reveals a change in secretion of some enzymes, when we monitor the genetic improvement of the strain. This can be evidenced by the xylanase activity, which activity increased nearly ten times when comparing the mutants S1M29 (this work) with 9A02S1 [4]. In smaller proportions, the same thing happens with β-glucosidases and cellobiohydrolases activities. In addition, the database employed in our work refers to the sequencing of *P. echinulatum* strains—another aspect that makes this proteomic analysis to be more consistent.

**Table 11:** Comparison between secretomic profiles of different *Penicillium echinulatum* strains

Comparative parameters	*Penicillium echinulatum* strains		
	Mutant 9A02S1 (Ribeiro et al. 2012)	Wild-type 2HH (This work)	Mutant S1M29 (This work)
Proteomic approach	Shotgun LC–MS/MS	Shotgun 1D-PAGE/LC–MS/MS	Shotgun 1D-PAGE/LC–MS/MS
Carbon sources	Sugar cane bagasse and cellulose	Sugar cane bagasse, cellulose, glucose, and glycerol	Sugar cane bagasse, cellulose, glucose, and glycerol
Days of growth	5	4	4
Proteins identified	99	147	129
Glycoside hydrolases	16	24	25
Major proteins found	Cellobiohydrolases Endoglucanases β-glucosidases Xylanases Debranching hemicellulose/pectin Pectinases Swollenin	Cellobiohydrolases Endoglucanases β-glucosidases Xylanases Debranching hemicellulose/pectin Pectinases Swollenin Cellulose monooxygenases Ligninases	Cellobiohydrolases Endoglucanases β-glucosidases Xylanases Debranching hemicellulose/pectin Pectinases Swollenin Cellulose monooxygenases Ligninases

The presence of cellulose monoxygenase Cel61A (Table 2/g3303), an important enzyme responsible for the oxidation of glycan chains on cellulose, found in *P. echinulatum* S1M29 secretome, is one more difference of this mutant; on the proteomic analysis of mutant strain 9A02S1, this key enzyme was not found. This feature also makes the secretome of *P. echinulatum* even more similar to *P. oxalicum*; besides presenting various cellulases and hemicellulases in common, this enzyme is found in higher proportions in *P. oxalicum* than in *T. reesei* [12].

The presence of nonhydrolytic proteins like swollenins, proteins similar to plant cell wall expansins, which can disrupt the crystalline parts of the cellulose chains, was observed only in the medium formulated with SCB or cellulose; for both strains, however, more were expressed in the mutant strain. Swollenins contain a carbohydrate-binding domain (CBM) and have been proposed to disrupt cellulose structure via nonhydrolytic mechanisms [44], although the biochemical action of these proteins remains to be fully elucidated [45]. As substrate accessibility is one key issue in plant cell wall degradation, accessory proteins are likely to enhance the efficiency of this process [46]. However, in some cases, the presence of a CBM in an enzyme may also prevent it from acting at different points on a substrate.

The presence of some enzymes involved in the degradation/depolymerization of lignin, such as isoamyl alcohol oxidase [47], manganese superoxide dismutase [48], and glutathione *S*-transferase [49] showed the first evidence of these enzymes’ production by the fungus *P. echinulatum.* This is more a characteristic of the genetic evolution of the fungus, since the secretome analysis of mutant 9A02S1 [5], strain before the S1M29 mutant, failed to identify lignin proteins, such as extracellular oxidase.

The lignin peroxidase, manganese-peroxidase, and laccase were generally considered as lignin degrading enzymes. However, according to Blanchette et al. [50] they are too big to penetrate the plant cell wall. Hence, to get access microbes initially activate easily diffusing several oxidases, reactive radical generating enzymes, and quinine reducing enzymes [51, 52]. The spectral counts of isoamyl alcohol oxidase, flavin adenine dinucleotide (FAD) oxidoreductase, and superoxide dismutase indicated that *P. echinulatum* degrade lignin through oxidases. In addition, this study also quantified expressions of glutathione-*S*-transferase, another enzyme important to the mechanism of lignin degradation.

The results obtained in this work suggest that in the genetic improvement process of *P. echinulatum*, performed by mutagenesis employing different mutagens and protoplast fusions [15, 16], numerous changes, possibly at the level of regulation/gene expression, post-translational modifications, and alterations in the capacity to secrete extracellular proteins, occurred with the mutant. These doubts will be elucidated in the future with a conjunct analysis of the secretome, transcriptome, and genome data.

## Conclusions

In this study, among the 165 proteins identified in the secretomes of both strains, approximately 40 % were proteins with CAZy function. The glycoside hydrolases were the most abundant. The secretome of *P. echinulatum* presents a potential for biomass degradation, mainly of cellulose and hemicellulose. Pectinases were found in minor amounts; however, for the first time, proteins related to lignin degradation were found.

Although cellulose and SCB were the carbon sources resulting in the production of cellulolytic and hemicellulolytic enzymes, glucose and glycerol inducing other proteins as enzymes related to lignin degrading and the presence of β-glucosidase were found more in glycerol than in any other carbon source. Differences in the protein expressions through enzymatic activity and by analyzing the number of spectra generated by proteomics data show the distinction between the secretomes of wild-type 2HH and mutant S1M29, in which the mutant toolbox is more focused on the production of cellulases and hemicellulases than the wild type. These findings allow us to confirm the potential that the strains of *P. echinulatum*, in particular, the mutant, have for the lignocellulosic biomass degradation.

## Methods

### Growth and maintenance of *Penicillium* strains

The wild-type 2HH and mutant S1M29 strains of *P. echinulatum* were grown and maintained in 100 mL of cellulose agar (agar-C) consisting of 40 mL of swollen cellulose, 10 mL of mineral solution (KH_2_PO_4_, 10 g; MgSO_4_.7H_2_O, 6 g; CO(NH_2_)_2_, 6 g; CaCl_2_, 6 g; FeSO_4_.7H_2_O, 0.1 g; MnSO_4_.H_2_O, 0.0312 g); ZnSO_4_.7H_2_O, 0.028 g; CoCl_2_.6H_2_O, 0.04 g; 1 L distiller water), 0.1 g of proteose peptone (Oxoid L85^®^), 2 g of agar, and 50 mL of distilled water. The strains were grown in inclined tubes on C-agar for 7 days at 28 °C until the formation of conidia, and then stored at 4 °C, as shown in Dillon et al. [15].

### Genome sequencing, assembly, and scaffolding

#### Genome sequencing

High-molecular-weight genomic DNA was extracted from both the 2HH and S1M29 strains using a protocol for DNA isolation [53] and used to generate libraries for Illumina Sequence by Synthesis (Illumina-SBS) genome sequencing using an unmodified Illumina TruSeq DNA protocol [54]. Post-adaptor ligation size-selected fragments were used for flow-cell cluster generation on the Illumina HiSeq 2000 platform. Illumina 100 bp paired-end chemistry sequencing was conducted using the commercial provider Ambry Genetics (Aliso Viejo, CA, USA). Illumina sequencing yielded 339,864.496 (33.98 GB) quality filtered reads for strain 2HH and 272,677.192 (27.26 GB) for strain S1M29 respectively.

#### Genome assembly

Illumina quality filtered reads from both the 2HH and S1M29 genome were assembled separately using the De-Bruijn graph short read assembly program Velvet version 1.2.10. The assembly run-time settings used for Velvet were a kmer value of 47, a minimum contig coverage of 7× and a minimum contig length setting of 300 bp. The Velvet assembly produced a total of 29.82 MB, 1018 contigs (n50 147.866 bp/n90 32.016 bp) for the 2HH genome and 29.83 MB, 1124 contigs (n50 137.718/n90 32.016) for the S1M29 genome, respectively. All assembled contigs were used for subsequent gene model creation, functional annotation, comparative studies, and downstream analysis.

#### Gene calling

All contigs produced from both Velvet assemblies of S1M29 and 2HH were used for genomic analysis. A total of 392,882,306 RNA-seq cDNA reads (39.28 GB) were generated from S1M29 strain using the Illumina TruSeq protocol with poly-A selection were used for gene model training prediction and cDNA alignment using the Eukaryotic gene prediction software Augustus. RNA-seq data were supplied as training files as mentioned by the Augustus RNA-seq protocol which leverages the PASA (Program to Assemble Spliced Assemblies) software [55]. Gene models produced by the Augustus protocol were used for genome annotation, proteomic database creation, and comparative analysis.

#### Functional annotation and proteome data bank assembly

Bioinformatics tools were used to build a secretome table. To validate the proteomic data the Scaffold 4 Proteomic software (version 4.3.2 20140225) was employed, where the database of proteins from the two strains of *P. echinulatum* (2HH and S1M29) were crossed. Conditions configured to accept the identification of a protein were: protein probability thresholds greater than 99 %, with a minimum of two different peptides for protein identification, each one with 95 % certainty. The search results showed false discovery rate (FDR) of peptides and proteins equal to zero.

Spectrum count was introduced as a semiquantitative method for the analysis of shotgun mass spectrometry data. Spectrum counts are the total number of tandem spectra assigned to each protein and are commonly used to determine their relative protein abundances, since previous studies have demonstrated a linear correlation between the spectrum count and protein abundance in complex samples [38].

As was used in the analysis performed using the methodology of spectrum count, the FDR was used as one of the parameters for determining the reliability of our experiments. The FDR is defined as the expected percentage of accepted peptide-spectrum match (PSM), with an associated score that is incorrect, where an “accepted PSM” is when it scores above the threshold [56]. Initially, a score is given for each peptide in the primary analysis performed with the Mascot Distiller software v.2.3.2.0, 2009 (Matrix Science Ltd.). This value shows the abundance of each peptide and also the reliability of these data. When the analysis was performed with the Scaffold 4 Proteomic software, the number of spectra was used to measure the abundance and the FDR for reliability. The threshold of ion score was 25 and, as mentioned before, our results showed FDRs of peptides and proteins are equal to zero.

In order to verify if the sequences of proteins annotated from the genomes of 2HH and S1M29 strains with different identification were the same, the alignment of the proteins was performed using the Clustal Omega software (Multiple Sequence Alignment—EMBL-EBI) [57].

To verify the protein sequences, BLAST (basic local alignment search tool) [58] software was employed. The parameters used to make the choice of protein were: *e* value ≤ e^−40^; identity ≥ 40 % and query cover ≥ 80 %.

With the aim of finding proteins with activity on carbohydrates (CAZymes), as well as the presence or absence of carbohydrate-binding modules, the software dbCAN (DataBase for Carbohydrate-active enzyme ANotation) was used [59].

The identification of secreted proteins was defined by the interpretation of the results of the following softwares, SignalP (version 4.1, Server-CBS) [60], SecretomeP (version 2.0) [61] and YLoc (interpretable subcellular localization prediction) [62].

### Production of enzymes

The culture medium for enzyme production consisted of 0.2 % (*w/v*) peptone; 0.05 % (*w/v*) Prodex^*®*^; 1 % (*w/v*) carbon source; 0.1 % (*v/v*) Tween 80^®^; 0.002 % (*v/v*) antibiotic ciprofloxacin (Proflox^®^, EMS S/A); 5 % (*v/v*) mineral solution (describe in “Growth and maintenance of *Penicillium* strains”); and distilled water to complete a final volume of 100 mL.

Erlenmeyer flasks (500 mL), containing 100 mL of the production medium, were inoculated with a suspension of 1 × 10^5^ conidia per mL and kept at 28 °C, in a reciprocal agitation of 180 rpm for 120 h. The experiment was performed in triplicate, and supernatants for 48, 96, and 120 h of cultivation were separated for protein determination, and the 96-h one was also used for proteomic data and enzymatic analysis. The samples were kept under refrigeration and sodium azide for a final concentration of 0.02 % (*w/v*).

To evaluate the effect of different carbon sources on the production of enzymes by the wild-type and mutant *P. echinulatum*, the following were employed: cellulose *Celuflok* E^®^ (Celuflok Comercial Ltda, Cotia, SP, Brazil), sugar cane bagasse pretreated by steam explosion (Usina Vale do Rosário, Morro Agudo, SP, Brazil), glucose (Quimidrol, Joinville, SC, Brazil), and glycerol (Sigma-Aldrich, St. Louis, MO, USA).

### Enzyme dosages

The specific enzymatic activity of the collected supernatants at 96 h of cultivation point was tested using a set of 13 different substrates (Megazyme, Wicklow, Ireland; and Sigma-Aldrich, St. Louis, MO, USA) in order to measure the activity of cellulases, hemicellulases, pectinases, esterases, and amylases.

To determine the cellulases, the following substrates were employed: Avicel^®^, carboxymethyl cellulose (CMC), lichenan (from *Cetraria islandica*), *p*-nitrophenyl-β-d-cellobioside (pNPC), and *p*-nitrophenyl-β-d-glucopyranoside (pNPG). The following substrates were employed to determine the hemicellulases: rye arabinoxylan, xylan from beechwood, *p*-nitrophenyl-β-d-xylopyranoside (pNPX), and mannan. Pectinases were measured using pectin substrate (from citrus fruits) and p-nitrophenyl-α-d- arabinofuranosidase (pNPA). Esterases were determined with feruloyl acetate and amylases were dosed with potato starch substrate.

All enzymatic activities were performed in triplicate. The methodology employed to determine enzymatic activities was performed according to Cota et al. [63] with modifications, where 50 μL of substrate solution (0.5 % *w/v* of the substrate diluted in water) were added to 46 μL of enzyme solution and 4 μL of sodium citrate buffer 1 mol/L, pH 4.8. The mixture was incubated at 50 °C for different periods depending on the substrate. The duration of the reaction was 30 min for potato starch, Avicel^®^, CMC, mannan, pectin, pNPA, pNPX, pNPC, and pNPG (for the latter two, 20 μL of enzyme solution, 30 μL of sodium citrate buffer 1 mol/L dilute to the samples containing cellulose or SCB). The reaction time of 10 min was used for lichenan, for samples containing SCB or cellulose (10 μL enzyme solution, 4 μL sodium citrate buffer, and 36 μL distilled water); 10 min for xylan and rye arabinoxylan for samples of the mutant strain grown in SCB or cellulose (10 μL enzyme solution, 4 μL sodium citrate buffer, and 36 μL distilled water). The reactions with polysaccharides were stopped by adding 300 and 100 μL of dinitrosalicylic acid reagent solution (DNS), respectively [64]. The reactions carried out with pNP substrates were stopped by adding 100 μL of sodium carbonate 10 (*w/v*) [65].

The activity of esterases was determined according to the methodology described by Koseki et al. [66]. For the samples containing cellulose or SCB, 5 μL of enzyme solution and 20 μL of sodium acetate buffer (SAB) 100 mmol/L, pH 5.5 were used, and 15 μL distilled water was added. For samples containing glucose or glycerol as carbon sources, 20 μL of enzyme solution and 20 μL of SAB buffer were used. Then, 10 μL of substrate (50 mmol/L in dimethylsulfoxide solution, DMSO) was added, incubated at 40 °C in a thermocycler for 30 min. Next, the reaction was terminated by adding 100 μL solution of Fast Garnet reagent (0.1 % *w/v* and SDS 15 % *w/v* in DMSO) and kept at room temperature for 10 min.

The units of enzymes with activity on the polysaccharides were defined as the amount of enzyme capable of releasing 1 μmol of reducing sugar per min. Units of enzyme with activity on the *p*-nitrophenyl substrates were defined as the amount of enzyme capable of liberating 1 μmol of *p*-nitrophenyl per min. Esterases were defined as the amount of enzyme capable of liberating 1 μmol of α-naphthyl acetate per minute.

### Determination of total soluble proteins

For the quantitative determination of soluble proteins in the secretomes of *P. echinulatum* wild-type and mutant strains, we employed the method of Bradford [67] with Bio-Rad Protein Assay. A calibration curve was constructed using 80 μL bovine serum albumin (BSA) standard solutions with concentrations between 0 and 25 μg/mL and 20 μL of Bradford reagent. The protein quantification of *P. echinulatum* secretome samples was carried out with 20 μL of Bradford reagent and 80 μL of sample. The reaction was incubated for 10 min at room temperature, and then readings were taken on a spectrophotometer at 595 nm.

### SDS-PAGE of total proteins

Samples collected at 96 h of cultivation from *P. echinulatum* were subjected to analysis of the protein profile. Samples containing cellulose or SCB were made in biological triplicate, whereas the samples containing glucose or glycerol were produced as a replicate. The most relevant comparisons are between the inducing sources, cellulose and SCB. Glucose and glycerol were used as external controls of low production, somehow, serving as a kind of normalization of experiments.

To determine the molecular weight of proteins, electrophoresis was performed in polyacrylamide gels containing 0.1 % (*w/v*) sodium dodecyl sulfate (SDS-PAGE). The separating gel was prepared in 12 % (*w/v*) while the stacker gel was prepared 4 % (*w/v*) according to the methodology described by Laemmli [68]. After standard the samples in 11 μg, it was applied to each slot to electrophoresis running in vertical cube Bio Rad Mini Protean System Cell at 110 V for approximately one and a half hours.

The revelation of bands from the gel was performed incubating the gel for 30 min in a solution of 0.2 % (*w/v*) Coomassie Brilhant Blue G 250, 50 % (*v/v*) of ethanol, and 10 % (*v/v*) of acetic acid. Afterward, the gel was washed with distilled water and immersed in a solution of 50 % (*w/v*) ethanol and 10 % (*w/v*) acetic acid for 30 min. The entire process was performed under stirring of 50 rpm until bands were visualized.

### Digestion of proteins for analysis by mass spectrometry

The digestion of proteins for analysis by mass spectrometry was performed in two steps, performing them in two successive days, according to Gonçalves et al. [69]. First we cut eight gel bands per lane. SDS was removed with 500 μL of a destain solution during 2 h and the bands were dehydrated for 5 min with 200 μL acetonitrile, reduced for 30 min with 30 μL dithiothreitol (DTT) solution, and alkylated with 30 μL of iodoacetoamide (IAA) solution, also for 30 min. Then the bands were washed with 100 mmol/L ammonium bicarbonate (for 10 min). A new dehydration with acetonitrile and rehydration with sodium bicarbonate was performed. Protein digestion was carried out with 30 μL of trypsin solution (1 mg/mL) in 50 mmol/L ammonium bicarbonate at 37 °C overnight.

On the second day, 10–30 μL of extraction solution 1 (5 % (*v/v*) of formic acid) was added to each microtube (depending on the size of the gel), incubated for 10 min at room temperature, given a quick spin in a centrifuge, and then the supernatant was collected and transferred to another microtube. Then, 12 μL of the second extraction solution (5 % (*v/v*) formic acid in 50 % (*v/v*) acetonitrile) was added to each microtube, and after 10 min, the supernatant was collected and transferred to a tube which was previously kept separated and had already contained the extract from the previous step. This step was repeated once more. Finally, the samples were evaporated in the speed vac, and approximately 1 μL of sample was left. The samples were stored at −20 °C until being transferred to the mass spectrometer subsequently.

### Liquid chromatography-tandem mass spectrometry

For the application of trypsinized fractions in the LC–MS/MS, each sample was resuspended with 12 μL of 0.1 % (*v/v*) formic acid and aliquot of 4.5 μL of the peptides mixture was injected into the chromatograph RP-nanoUPLC (nanoAcquity, Waters). Chromatography was performed on C18 (100 × 100 mm) equilibrated with 0.1 % (*v/v*) formic acid column buffer. The elution gradient was 2 % (*v/v*) to 90 % (*v/v*) acetonitrile in 0.1 % (*v/v*) formic acid. The whole system operated at the speed of 0.6 µL/min. As the peptides were eluted from the column, they were injected into the spectrometer quadrupole-time of flight Q-Tof (Ultima Mass Spectrometer Waters) with a source of ionization electron spray, for 60 min. The instrument was operated in the “top three–MS and MS/MS mode”, where each MS spectrum acquired, its three peptides not monoloaded (precursors) most abundant were selected for further fragmentation (generating y and b sets) and sequenced generating a MS/MS spectrum for each peptide.

The spectra were acquired using MassLynx v.4.1 software (Waters, Milford, MA, USA), and the raw data were converted to the format “peak list format (mgf)” by Mascot Distiller software v.2.3.2.0, 2009 (Matrix Science Ltd.). These results were processed by Mascot v.2.3.01 engine (Matrix Science Ltd.) software against the genome-sequencing database of *P. echinulatum* strains (2HH: 8504 sequences, 4286814 residues; M29: 8552 sequences, 4298942 residues) for digestion and theoretical spectral generation of MS/MS. The following parameters were used in this process: carbamidomethylation as a fixed modification, oxidation of methionine as a variable modification, one error of trypsin cleavage, and maximum allowable error in the peptide mass of 0.1 Da. Only peptides with at least five amino acids and a score within probability to be not a random event *p* < 0.05 were selected as a peptide cleavage product (i.e., they are part of a protein). The peptide was considered only when it differed by at least one amino acid for another or when it differed in covalent modifications (including elongations of N- or C-terminal).

### Analysis of results

The analyses of data regarding the protein concentration and enzymatic activities were performed in PrismGraphPad (version 5.0.1.334) software. The statistical analysis of protein concentration was performed by analysis of variance and Tukey’s post-test for a *p* < 0.05, and the statisticSpecific enzymatic activity of Penicilliumal analysis of spectra was performed by *t* test for a *p* < 0.05.

## Additional files

10.1186/s13068-016-0476-3 Other groups of proteins identified and spectrum count in different carbon sources by wild type 2HH and mutant S1M29 of *Penicillium echinulatum* at 96 h during submerged cultivation. This table shows other groups of proteins found in *Penicillium echinulatum* secretome, as adhesion proteins, oxidoreductases, proteases and peptidases, lipases, glutaminases, chaperones, hypothetical proteins and miscelaneous proteins.
10.1186/s13068-016-0476-3 Comparative analysis of the most expressed proteins in each condition for each *Penicillium echinulatum* strain. This comparative analysis was based on the list, in order, of ten proteins with more spectra found in each carbon source in the wild type 2HH and mutant S1M29 and also the most expressed proteins when analyzing both strains.
10.1186/s13068-016-0476-3 Complete protein reports of *Penicillium echinulatum* strains in each condition. This table shows important data of proteomic analysis: number of peptides used for the identification and sequence coverage (%).

## Abbreviations

1D-PAGE: one dimensional polyacrylamide electrophoresis
LC–MS/MS: liquid chromatography-tandem mass spectrometry
CAZy/CAZymes: carbohydrate active enzymes
SCB: sugar cane bagasse pretreated by steam explosion
CEL: cellulose
GLY: glycerol
GLU: glucose
GHs: glicoside hydrolases
CEs: carbohydrate esterases
PLs: polysaccharide lyases
AAs: auxiliary activities
CBMs: carbohydrate-binding module
pNP: *p*-nitrophenyl
pNPC: *p*-nitrophenyl-β-d-cellobioside
pNPG: *p*-nitrophenyl-β-d-glucopyranoside
pNPX: *p*-nitrophenyl-β-d-xylopyranoside
pNPA: *p*-nitrophenyl-α-d-arabinofuranoside
AVI: Avicel^®^
LIC: lichenan
CMC: carboxymethyl cellulose
ARAX: rye arabinoxylan
XIL: xylan
MAN: mannan
PEC: pectin
FEA: feruloyl acetate
PST: potato starch
DNS: dinitrosalicylic acid
SAB: sodium acetate buffer
DMSO: dimethyl sulfoxide
BSA: bovine serum albumin
DTT: dithiothreitol
IAA: iodoacetoamide
SDS: sodium dodecyl sulfate
UPLC: ultra performance liquid chromatography
Q-Tof: quadrople time of fligth
FDR: false discovery rate
PSM: peptide-spectrum match
BLAST: basic local alignment search tool
dbCAN: database for carbohydrate-active enzyme annotation
YLoc: interpretable subcellular localization prediction
