# Supplementary material for: Penicillium echinulatum secretome analysis reveals the fungi potential for degradation of lignocellulosic biomass
Source: Biotechnol Biofuels. 2016 Mar 17;9:66. doi: 10.1186/s13068-016-0476-3 (PMC4794826; doi:10.1186/s13068-016-0476-3)
Supplement: Supplementary file 1 — 10.1186/s13068-016-0476-3 Other groups of proteins identified and spectrum count in different carbon sources by wild type 2HH and mutant S1M29 of Penicillium echinulatum at 96 h during submerged cultivation. This table shows other groups of proteins found in Penicillium echinulatum secretome, as adhesion proteins, oxidoreductases, proteases and peptidases, lipases, glutaminases, chaperones, hypothetical proteins and miscelaneous proteins. [file 13068_2016_476_MOESM1_ESM.docx]

**Additional file 1: Other groups of proteins identified and spectrum count in different carbon sources by wild type 2HH and mutant S1M29 of *Penicillium echinulatum* at 96 h during submerged cultivation.**

| **Accession**  **Number** | **Identified proteins** | **Organism** | | **MW (kDa)** | **Secretion^b^** | **Spectrum count^a^** | | | | | | | | |
| --- | --- | --- | --- | --- | --- | --- | --- | --- | --- | --- | --- | --- | --- | --- |
|  |  |  |  |  |  | **2HH** | | | | **S1M29** | | | | |
|  |  |  |  |  |  | **Glu** | **Gly** | **SCB** | **Cel** | **Glu** | **Gly** | **SCB** | **Cel** | |
| ***Adhesion proteins*** | | | | | | | | | | | | | | |
| g5908 | Filamentous hemagglutinin/adhesin | *Beauveria bassiana* | | 26 | Y | 23 | 21 | 18 | 40 | 0 | 15 | 8 | 5 | |
| g1204 | GPI anchored cell wall protein | *Neosartorya fischeri* | | 23 | Y | 39 | 8 | 4 | 6 | 11 | 10 | 2 | 6 | |
| g4263 | GPI anchored cell wall protein | *Aspergillus oryzae* | | 34 | Y | 0 | 18 | 7 | 13 | 0 | 3 | 0 | 0 | |
| g8160 | GPI-anchored cell wall protein | *Aspergillus ruber* | | 42 | Y | 8 | 1 | 2 | 1 | 4 | 8 | 0 | 3 | |
| g120 | Mannose-binding lectin | *Penicillium roqueforti* | | 50 | Y | 0 | 0 | 0 | 0 | 0 | 15 | 0 | 3 | |
| g5107 | IgE-binding protein | *Aspergillus oryzae* | | 24 | N | 18 | 5 | 2 | 9 | 5 | 0 | 17 | 0 | |
| g5607 | IgE binding protein | *Penicillium digitatum* | | 23 | Y | 0 | 0 | 0 | 0 | 42 | 0 | 0 | 0 | |
| ***Oxidoreductases*** | | | | | | | | | | | | | | |
| g1768 | D-xylose reductase | *Aspergillus fumigatus* | | 36 | N | 0 | 0 | 80 | 23 | 0 | 0 | 15 | 8 |  |
| g992 | Malate dehydrogenase, mitochondrial precursor | *Aspergillus terreus* | | 36 | N | 0 | 7 | 2 | 10 | 0 | 0 | 0 | 0 | |
| g2710 | Nitroreductase family protein | *Penicillium digitatum* | | 29 | N | 0 | 0 | 9 | 0 | 0 | 0 | 1 | 0 |  |
| g5742 | Manganese superoxide dismutase | *Penicillium chrysogenum* | | 20 | N | 0 | 10 | 0 | 8 | 0 | 3 | 0 | 1 | |
| g6048 | Formate dehydrogenase | *Byssochlamys spectabilis* | | 45 | N | 0 | 6 | 0 | 3 | 0 | 0 | 0 | 0 | |
| g5493 | Cu,Zn superoxide dismutase SOD1 | *Neosartorya fischeri* | | 18 | Y | 0 | 0 | 41 | 0 | 0 | 0 | 0 | 0 | |
| g7218 | Mannitol-1-phosphate 5-dehydrogenase | *Penicillium roqueforti* | | 44 | Y | 0 | 1 | 0 | 3 | 0 | 0 | 0 | 0 | |
| g2794 | Aldehyde dehydrogenase | *Penicillium roqueforti* | | 54 | N | 0 | 2 | 0 | 0 | 0 | 0 | 0 | 0 | |
| ***Proteases and peptidases*** | | | | | | | | | | | | | | |
| g5859 | Aminopeptidase 2 | *Aspergillus terreus* | | 99 | N | 0 | 21 | 19 | 12 | 0 | 22 | 2 | 0 | |
| g6527 | Aspartic endopeptidase | *Aspergillus fumigatus* | | 43 | Y | 0 | 6 | 11 | 7 | 0 | 16 | 2 | 4 | |
| g3775 | Tripeptidyl peptidase A | *Neosartorya fischeri* | | 64 | Y | 28 | 0 | 3 | 0 | 87 | 0 | 0 | 4 | |
| g592 | Prolidase | *Penicillium digitatum* | | 52 | N | 0 | 2 | 9 | 2 | 0 | 15 | 0 | 1 | |
| g2787 | Yapsin | | *Talaromyces marneffei* | 53 | Y | 3 | 0 | 0 | 0 | 0 | 0 | 0 | 8 |  |
| g5496 | Vacuolar serine protease | *Penicillium oxalicum* | | 53 | Y | 0 | 1 | 0 | 2 | 0 | 6 | 0 | 4 | |
| g2636 | Carboxypeptidase CpyA/Prc1 | *Neosartorya fischeri* | | 64 | Y | 0 | 1 | 2 | 1 | 0 | 3 | 0 | 1 | |
| g7745 | Aspartyl aminopeptidase | *Penicillium digitatum* | | 56 | N | 0 | 7 | 4 | 6 | 0 | 6 | 0 | 0 | |
| g8551 | Tripeptidyl-peptidase sed1 | *Penicillium digitatum* | | 70 | N | 10 | 0 | 0 | 0 | 0 | 0 | 0 | 0 | |
| g2785 | Aspergillopepsin | *Neosartorya fischeri* | | 28 | Y | 16 | 0 | 0 | 0 | 0 | 0 | 0 | 6 | |
| g4035 | γ-glutamyltranspeptidase | *Penicillium roqueforti* | | 63 | Y | 0 | 2 | 0 | 1 | 0 | 2 | 0 | 0 | |
| g7131 | Probable dipeptidyl peptidase 3 | | *Penicillium roqueforti* | 79 | N | 0 | 1 | 2 | 1 | 0 | 1 | 0 | 0 |  |
| g6667 | Aminopeptidase | | *Penicillium digitatum* | 99 | N | 0 | 2 | 0 | 0 | 0 | 1 | 0 | 0 |  |
| g708 | Aspartic protease pepAc | *Aspergillus niger* | | 47 | Y | 3 | 2 | 0 | 0 | 0 | 0 | 0 | 0 | |
| g5823 | carboxypeptidase S1 | *Talaromyces marneffei* | | 52 | Y | 1 | 1 | 0 | 0 | 0 | 0 | 0 | 0 | |
| g989 | Probable leucine aminopeptidase 2 | *Penicillium roqueforti* | | 53 | Y | 0 | 1 | 0 | 0 | 0 | 1 | 0 | 0 | |
| g3656 | Serine peptidase | *Byssochlamys spectabilis* | | 58 | Y | 1 | 0 | 0 | 0 | 0 | 1 | 0 | 0 | |
| g2790 | Penicillopepsin | *Penicillium janthinellum* | | 41 | Y | 0 | 0 | 0 | 0 | 0 | 0 | 0 | 1 | |
| g5790 | Peptidase S10, serine carboxypeptidase | *Penicillium roqueforti* | | 63 | Y | 0 | 2 | 0 | 0 | 0 | 0 | 0 | 0 | |
| g4250 | α/β-hydrolase | *Penicillium roqueforti* | | 38 | Y | 0 | 0 | 0 | 0 | 0 | 0 | 0 | 1 | |
| g1378 | Peptidase S9, prolyl oligopeptidase | *Penicillium roqueforti* | | 81 | Y | 0 | 43 | 10 | 10 | 0 | 40 | 1 | 5 | |
| g4879 | Amidase | *Aspergillus oryzae* | | 64 | Y | 6 | 9 | 0 | 0 | 0 | 8 | 0 | 0 | |
| ***Lipases*** | | | | | | | | | | | | | | |
| g1946 | Lipase | *Penicillium roqueforti* | | 30 | Y | 0 | 1 | 0 | 2 | 0 | 39 | 4 | 19 | |
| g347 | Lysophospholipase phospholipase B | *Penicillium chrysogenum* | | 69 | Y | 34 | 24 | 2 | 2 | 33 | 7 | 1 | 5 | |
| g1420 | Lipase, class 2 | *Penicillium roqueforti* | | 29 | Y | 0 | 0 | 0 | 9 | 0 | 0 | 0 | 0 | |
| ***Glutaminases*** | | | | | | | | | | | | | | |
| g6338 | Glutaminase A | *Byssochlamys spectabilis* | | 75 | Y | 0 | 3 | 5 | 3 | 2 | 11 | 0 | 5 | |
| g6870 | Glutaminase | *Penicillium digitatum* | | 93 | Y | 0 | 2 | 0 | 19 | 0 | 4 | 0 | 0 | |
| g5109 | Glutaminase | *Aspergillus niger* | | 75 | Y | 0 | 0 | 0 | 0 | 0 | 0 | 0 | 2 | |
| ***Chaperones*** | | | | | | | | | | | | | | |
| g6709 | Heat shock protein | *Penicillium digitatum* | | 70 | N | 0 | 2 | 0 | 1 | 2 | 1 | 0 | 0 | |
| g7547 | Mitochondrial Hsp70 chaperone (Ssc70) | *Penicillium digitatum* | | 72 | N | 0 | 1 | 0 | 0 | 0 | 4 | 0 | 0 | |
| g4939 | Tubulin-specific chaperone Rbl2 | *Neosartorya fischeri* | | 13 | N | 0 | 0 | 0 | 0 | 4 | 0 | 0 | 0 | |
| g351 | ATP-dependent molecular chaperone HSC82 | *Arthroderma otae* | | 80 | N | 0 | 0 | 0 | 0 | 2 | 0 | 0 | 0 | |
| ***Hypothetical proteins*** | | | | | | | | | | | | | | |
| g3760 | Hypothetical protein PDE_06089 | *Penicillium oxalicum* | | 18 | Y | 27 | 26 | 0 | 3 | 5 | 5 | 0 | 11 | |
| g5020 | Hypothetical protein PDE_06797 | *Penicillium oxalicum* | | 25 | Y | 0 | 46 | 0 | 0 | 0 | 0 | 0 | 0 | |
| g7861 | Hypothetical protein PDE_02536 | *Penicillium oxalicum* | | 19 | Y | 3 | 2 | 0 | 2 | 0 | 7 | 0 | 1 | |
| g2722 | Hypothetical protein PDE_02180 | *Penicillium oxalicum* | | 123 | Y | 28 | 0 | 0 | 0 | 0 | 0 | 0 | 0 | |
| g8113 | Hypothetical protein PDE_01252 | *Penicillium oxalicum* | | 16 | Y | 0 | 26 | 43 | 0 | 0 | 5 | 0 | 0 | |
| g2258 | Hypothetical protein PDE_04474 | | *Penicillium oxalicum* | 33 | Y | 0 | 1 | 1 | 4 | 0 | 1 | 0 | 1 |  |
| g2829 | Hypothetical protein PDE_00925 | *Penicillium oxalicum* | | 22 | Y | 8 | 0 | 0 | 0 | 2 | 0 | 1 | 0 | |
| g2294 | Hypothetical protein PDE_00503 | *Penicillium oxalicum* | | 17 | Y | 0 | 21 | 0 | 0 | 0 | 4 | 0 | 0 | |
| g3357 | Hypothetical protein PDE_05699 | *Penicillium oxalicum* | | 109 | Y | 39 | 0 | 0 | 0 | 18 | 0 | 0 | 0 | |
| g1259 | Hypothetical protein PDE_03110 | *Penicillium oxalicum* | | 19 | Y | 0 | 0 | 0 | 14 | 3 | 0 | 0 | 30 | |
| g5510 | Hypothetical protein PDE_02988 | *Penicillium oxalicum* | | 34 | Y | 6 | 3 | 1 | 0 | 0 | 0 | 1 | 1 | |
| g6328 | Hypothetical protein PDE_01808 | *Penicillium oxalicum* | | 11 | N | 19 | 0 | 11 | 2 | 2 | 0 | 0 | 0 | |
| ***Miscelaneous proteins*** | | | | | | | | | | | | | | |
| g4278 | Ubiquitin-40S ribosomal protein | *Aspergillus oryzae* | | 18 | N | 7 | 0 | 13 | 13 | 93 | 0 | 0 | 30 | |
| g5678 | Extracellular serine protein | *Aspergillus fumigatus* | | 87 | Y | 0 | 18 | 4 | 2 | 0 | 0 | 5 | 3 | |
| g4533 | Nucleoside diphosphate kinase | *Penicillium roqueforti* | | 17 | N | 0 | 4 | 21 | 5 | 0 | 2 | 21 | 7 | |
| g5115 | 6-phosphogluconolactonase | *Aspergillus kawachii* | | 45 | Y | 0 | 13 | 0 | 4 | 0 | 11 | 0 | 0 | |
| g1883 | Glucose-6-phosphate isomerase | *Penicillium roqueforti* | | 62 | N | 0 | 2 | 3 | 5 | 0 | 1 | 0 | 0 | |
| g5494 | Transaldolase | *Aspergillus flavus* | | 35 | N | 0 | 2 | 2 | 3 | 1 | 0 | 0 | 2 | |
| g3663 | Aldose /Glucose-6-phosphate 1-epimerase | *Penicillium roqueforti* | | 50 | N | 0 | 2 | 3 | 1 | 0 | 3 | 0 | 1 | |
| g7606 | Aminotransferase, class V | *Byssochlamys spectabilis* | | 66 | N | 0 | 1 | 2 | 3 | 0 | 2 | 0 | 0 | |
| g2745 | 2,3-dihydroxybenzoate decarboxylase | *Penicillium roqueforti* | | 39 | N | 0 | 0 | 2 | 5 | 0 | 0 | 0 | 0 | |
| g2426 | Elongation factor 1 γ-domain | *Aspergillus oryzae* | | 46 | N | 0 | 14 | 2 | 17 | 0 | 14 | 0 | 0 | |
| g2400 | Actin | *Penicillium oxalicum* | | 37 | N | 0 | 2 | 7 | 69 | 3 | 0 | 0 | 0 | |
| g8382 | Cytidine deaminase | *Penicillium roqueforti* | | 15 | Y | 0 | 0 | 11 | 0 | 0 | 1 | 0 | 7 | |
| g7675 | Fumarylacetoacetase | *Penicillium roqueforti* | | 47 | N | 0 | 1 | 3 | 1 | 0 | 0 | 0 | 0 | |
| g1374 | Phytase | *Aspergillus nidulans* | | 57 | Y | 4 | 3 | 0 | 0 | 0 | 2 | 0 | 0 | |
| g50 | Ribonuclease T2 | *Penicillium roqueforti* | | 28 | Y | 0 | 1 | 0 | 0 | 0 | 3 | 0 | 2 | |
| g3158 | 5-methyltetrahydropteroyltriglutamate homocysteine S-methyltransferase | *Aspergillus clavatus* | | 86 | N | 0 | 0 | 5 | 0 | 0 | 0 | 0 | 0 | |
| g153 | HIT domain protein | *Aspergillus kawachii* | | 15 | Y | 0 | 0 | 6 | 4 | 0 | 0 | 0 | 0 | |
| g3795 | Glutathione S-transferase | *Penicillium digitatum* | | 26 | N | 0 | 0 | 3 | 3 | 0 | 0 | 1 | 0 | |
| g7872 | Enolase/allergen Asp F 22 | *Aspergillus clavatus* | | 47 | N | 0 | 5 | 0 | 0 | 0 | 2 | 0 | 0 | |
| g409 | Cyanate hydratase | *Aspergillus nidulans* | | 18 | N | 0 | 0 | 10 | 5 | 0 | 0 | 11 | 0 | |
| g760 | Cytochrome b5 | *Aspergillus oryzae* | | 14 | N | 0 | 0 | 4 | 2 | 0 | 2 | 0 | 3 | |
| g7391 | Nuclease PA3^b^ | *Cordyceps militaris* | | 36 | Y | 0 | 3 | 0 | 2 | 0 | 3 | 0 | 0 | |
| g2296 | Phosphoesterase | *Penicillium roqueforti* | | 120 | N | 0 | 0 | 1 | 0 | 0 | 3 | 0 | 0 | |
| g8122 | Necrosis- and ethylene-inducing protein 2 precursor | *Botrytis tulipae* | | 26 | Y | 0 | 0 | 0 | 0 | 0 | 1 | 0 | 2 | |
| g597 | Cyclophilin-like peptidyl prolyl cis-trans isomerase | *Aspergillus niger* | | 21 | Y | 0 | 4 | 0 | 17 | 0 | 0 | 0 | 0 | |
| g2491 | HAD superfamily hydrolase | *Aspergillus oryzae* | | 28 | N | 0 | 1 | 0 | 5 | 0 | 0 | 0 | 0 | |
| g7753 | Inorganic pyrophosphatase | *Penicillium roqueforti* | | 33 | N | 0 | 1 | 0 | 5 | 0 | 0 | 0 | 0 | |
| g6008 | Purine nucleoside permeasse | *Penicillium roqueforti* | | 43 | N | 1 | 0 | 0 | 0 | 3 | 0 | 0 | 0 | |
| g4614 | Pyridoxine biosynthesis protein pyroA | *Penicillium digitatum* | | 32 | N | 0 | 1 | 0 | 0 | 0 | 3 | 0 | 0 | |
| g608 | Allergen Asp F4 | *Neosartorya fischeri* | | 36 | Y | 0 | 0 | 0 | 1 | 1 | 0 | 0 | 0 | |
| g371 | Actin-binding, cofilin/tropomyosin type | *Penicillium roqueforti* | | 50 | N | 0 | 0 | 0 | 0 | 0 | 0 | 0 | 1 | |
| g1092 | Conidial hydrophobin Hyp1/RodA | *Aspergillus kawachii* | | 15 | Y | 0 | 0 | 0 | 5 | 0 | 0 | 0 | 0 | |
| g4522 | Conidial pigment biosynthesis oxidase Arb2/brown | *Aspergillus oryzae* | | 67 | Y | 0 | 2 | 0 | 0 | 0 | 0 | 0 | 0 | |
| g7764 | Glutamine amidotransferase-like protein, class I | *Sphaerulina musiva* | | 26 | Y | 0 | 6 | 0 | 0 | 0 | 0 | 0 | 0 | |
| g7393 | Putative DNA repair exonuclease SIA1 | *Aspergillus oryzae* | | 46 | N | 0 | 0 | 0 | 0 | 0 | 1 | 0 | 0 | |
| g3371 | Actin-binding, cofilin/tropomyosin type | *Penicillium roqueforti* | | 25 | Y | 0 | 0 | 0 | 5 | 0 | 0 | 0 | 0 | |
| g8197 | 60S ribosomal protein L22 | *Talaromyces stipitatus* | | 14 | N | 0 | 0 | 0 | 0 | 2 | 0 | 0 | 0 | |
| g8526 | Dynein light chain | *Aspergillus niger* | | 15 | Y | 0 | 0 | 0 | 0 | 2 | 0 | 0 | 0 | |
| g1652 | Catalase B | *Penicillium roqueforti* | | 80 | Y | 0 | 0 | 0 | 0 | 0 | 1 | 0 | 0 | |

^a^Secretome analysis based on spectral counting. A quantitative analysis was conducted for samples grown on cellulose or SCB (mean of triplicates), while a semiquantitative analysis for samples grown on glucose or glycerol (one replicate) was performed.

^b^The secretion of each protein was verified by the softwares SignalP, SecretomeP and YLoc.
